# Supplementary material for: Alpha1A- and Beta3-Adrenoceptors Interplay in Adipose Multipotent Mesenchymal Stromal Cells: A Novel Mechanism of Obesity-Driven Hypertension
Source: Cells. 2023 Feb 11;12(4):585. doi: 10.3390/cells12040585 (PMC9954306; doi:10.3390/cells12040585)
Supplement: Supplementary file 1 [file cells-12-00585-s001.zip › Supplemental figures.pdf]

Normotensive obese patient

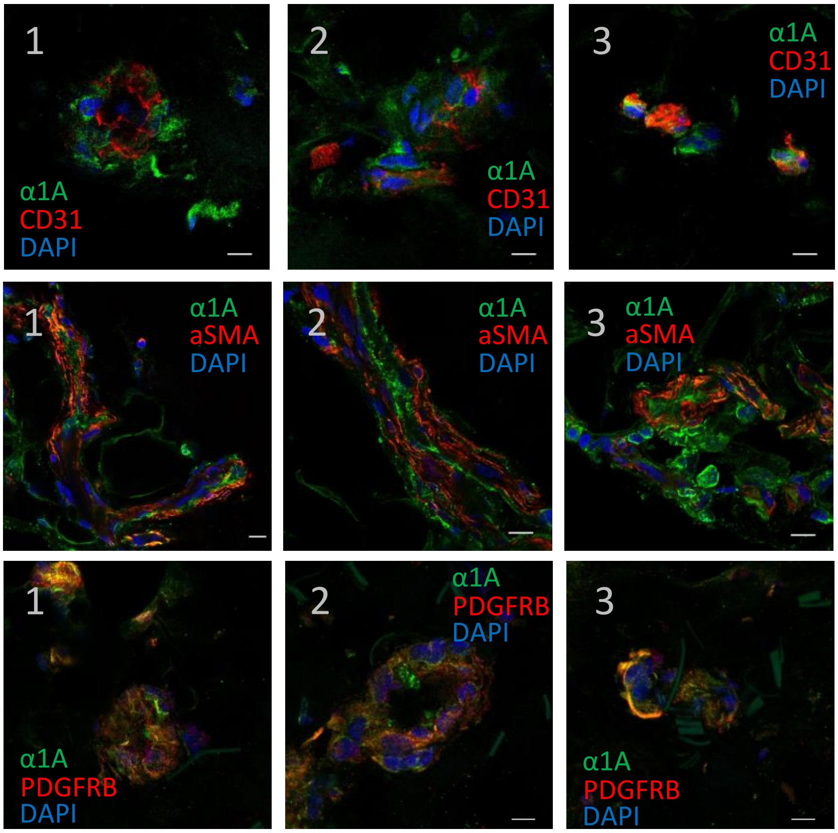

Hypertensive obese patient

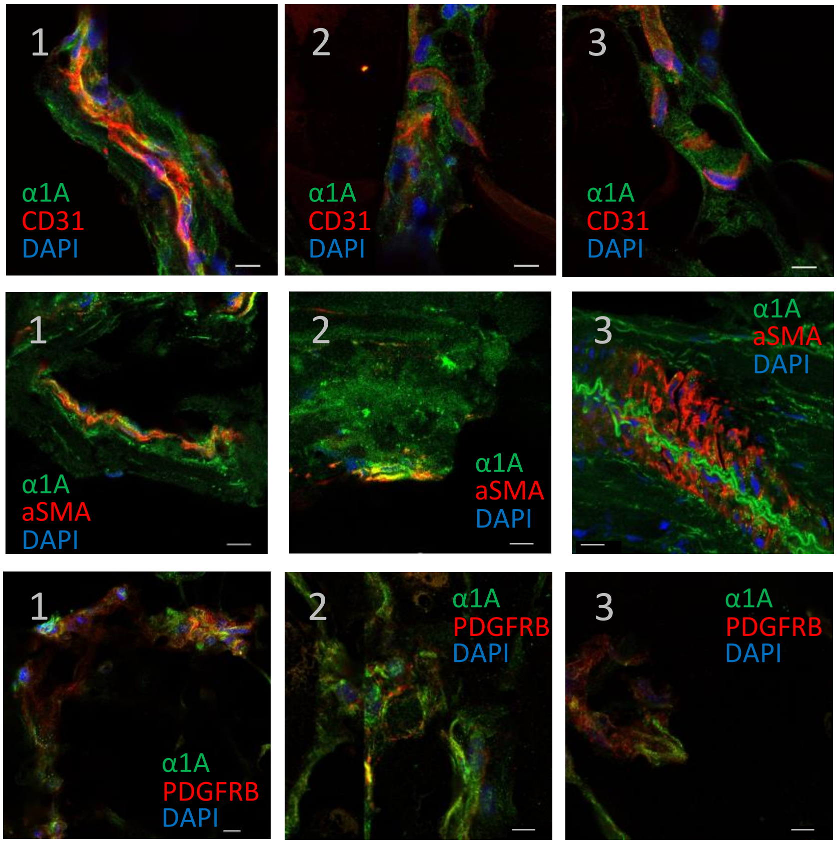

Figure S1. **Confocal images of frozen sections of human subcutaneous adipose tissue.** Cells expressing α1A-adrenergic receptor (green), CD31 positive endothelial cells (red), alpha smooth muscle actin+ cells (red), PDGFRβ+ perivascular cells (red). Nuclei stained with Dapi (blue). Scale bar 10 µm.

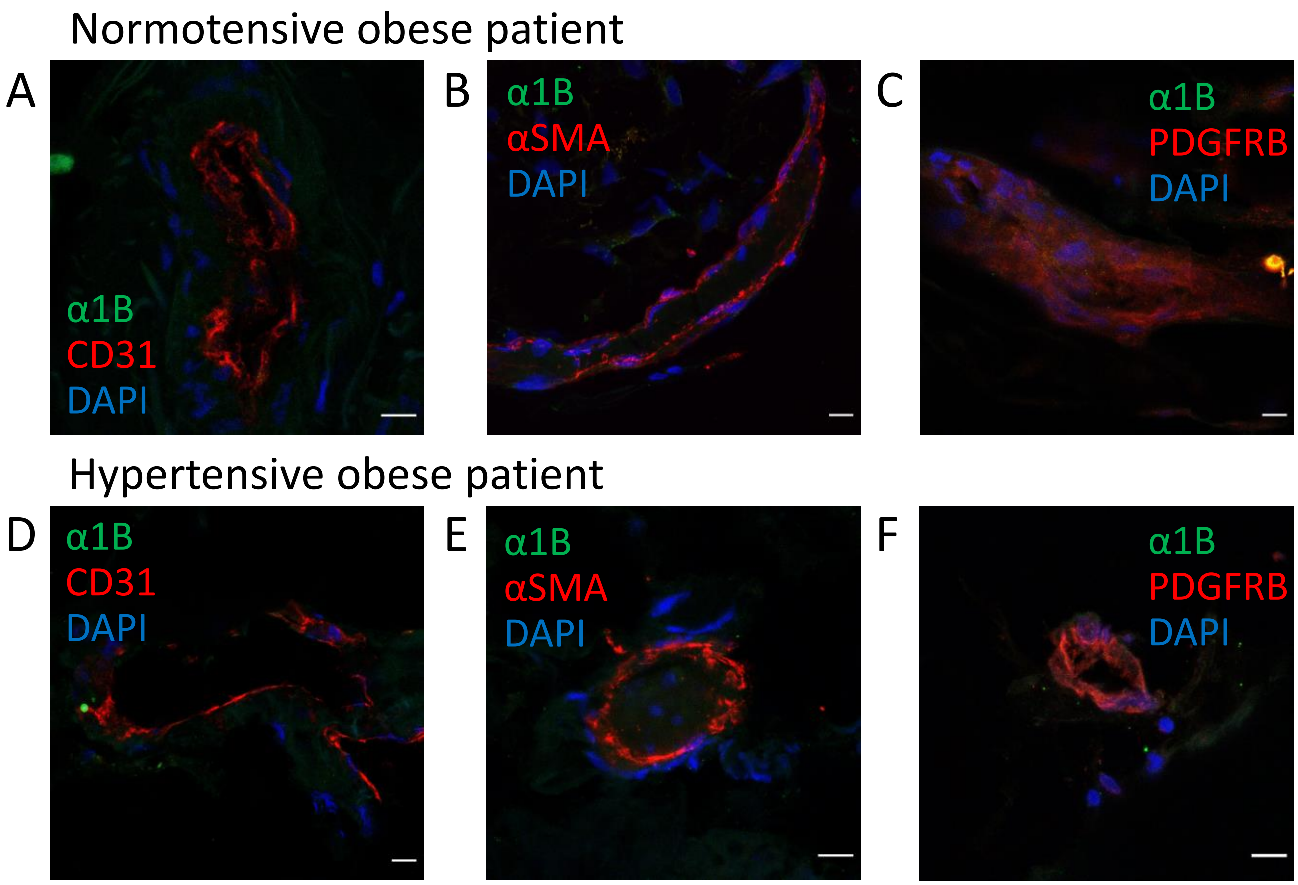

Figure S2. **Confocal images of frozen sections of human subcutaneous adipose tissue.** A-C, alpha1B-adrenoceptor expression in vessels of subcutaneous adipose tissue of obese normotensive patients. D-F, alpha1B-adrenoceptor expression in vessels of subcutaneous adipose tissue of obese hypertensive patients. Cells expressing α1B-adrenergic receptor (green), CD31 positive endothelial cells (red), alpha smooth muscle actin positive cells (red), PDGFRβ positive perivascular cells (red). Nuclei stained with Dapi (blue). Scale bar 10 µm.

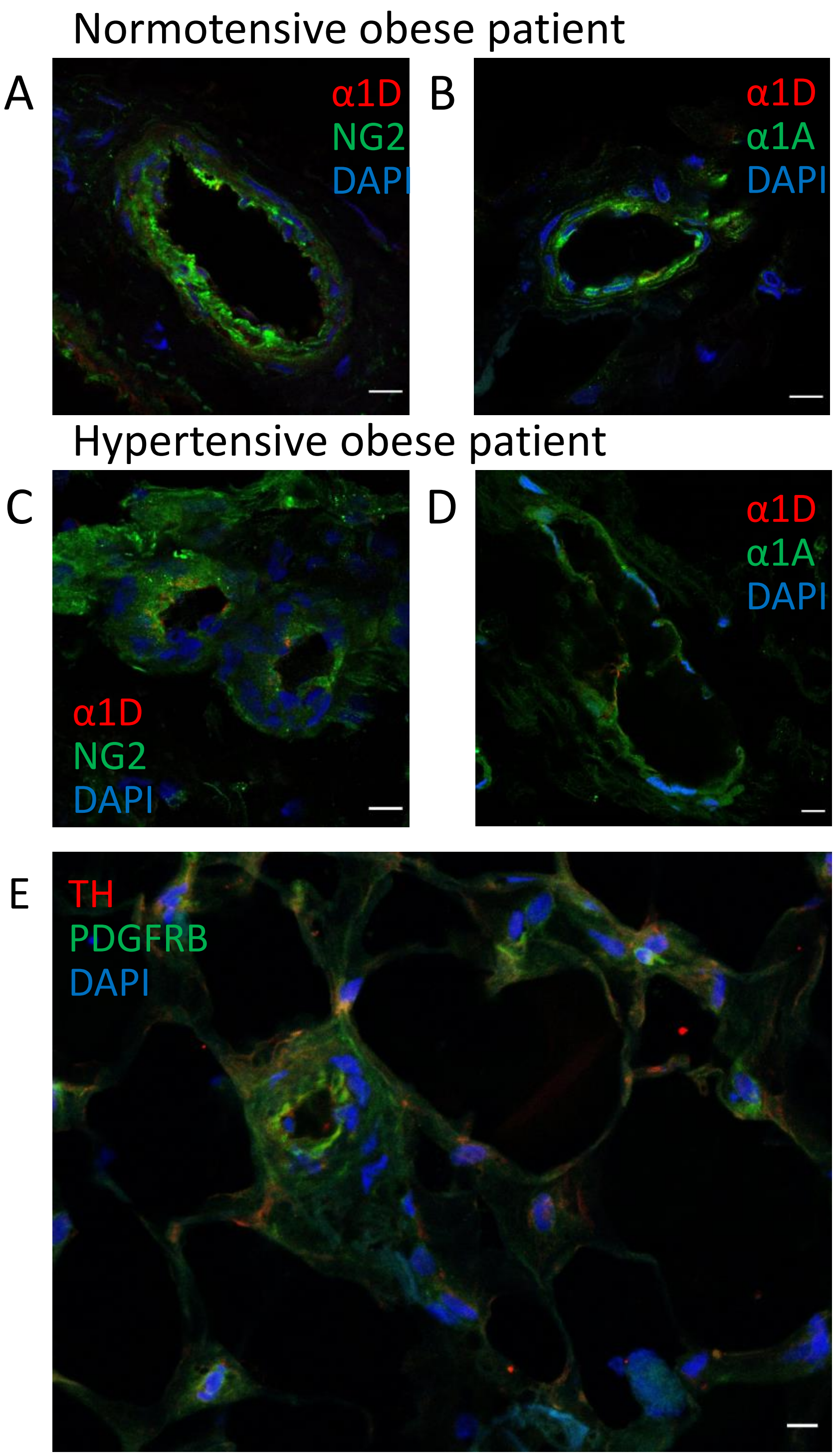

Figure S3. **Confocal images of frozen sections of human subcutaneous adipose tissue.** A-B, alpha1D-adrenoceptor expression in vessels of subcutaneous adipose tissue of obese normotensive patients. C-D, alpha1D-adrenoceptor expression in vessels of subcutaneous adipose tissue of obese hypertensive patients. E, Tyrosine hydroxylase positive sympathetic nerves in subcutaneous adipose tissue. Cells expressing α1D-adrenergic receptor (red), α1A-adrenergic receptor (green), NG2 positive pericytes (green), tyrosine hydroxylase (red), PDGFRβ+ perivascular cells (green). Nuclei stained with Dapi (blue).. Nuclei stained with Dapi (blue). Scale bar 10 µm.

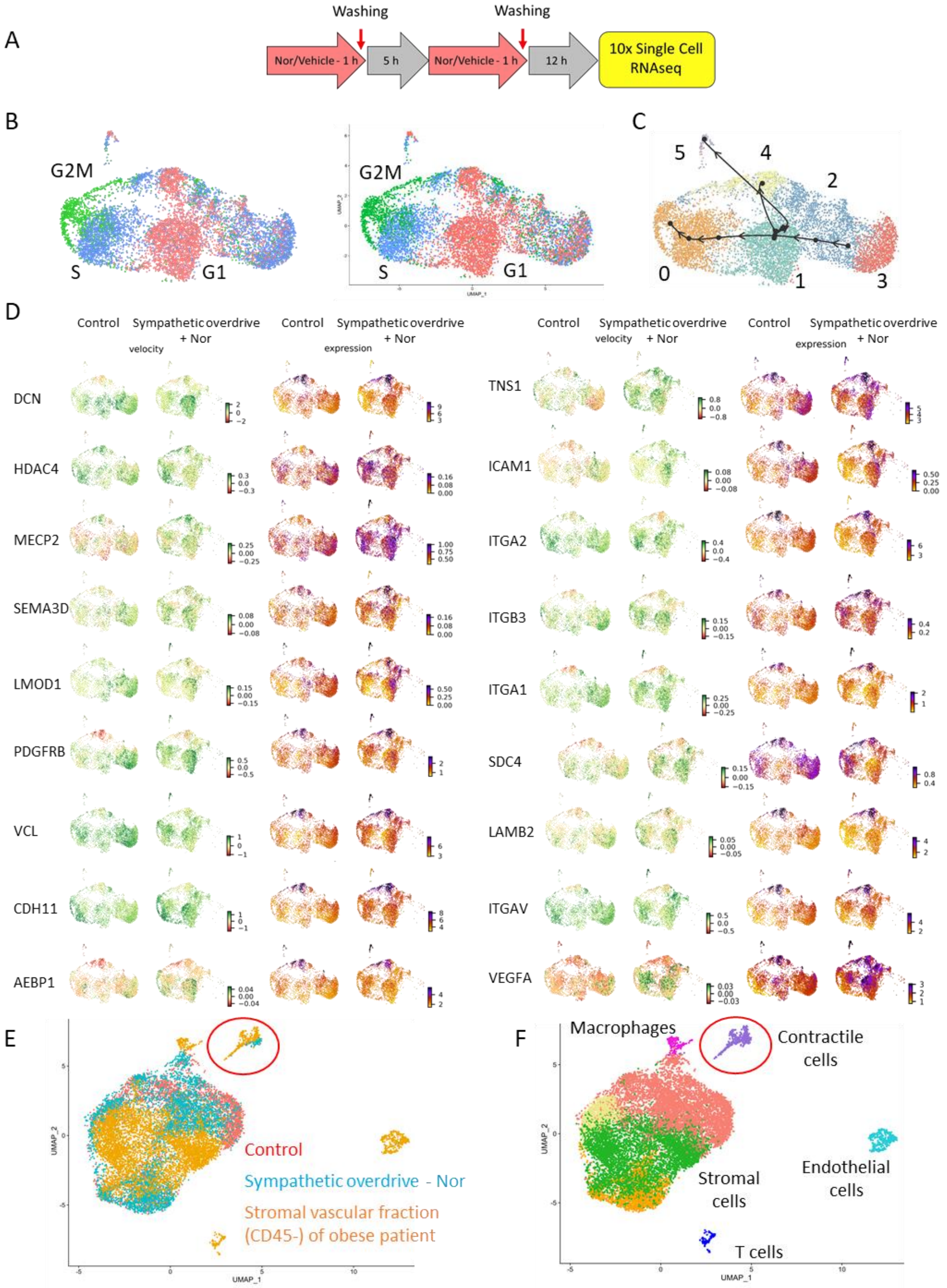

Figure S4. Single cell RNAseq of MSCs after sympathetic overdrive followed by noradrenaline treatment. A, Scheme of experiment of single cell RNAseq analysis of MSCs. B, G2M, S and G1 scores of cells in integrated data using scVelo (left) and Seurat (right). Dividing cells mainly localized in cluster 0. C, Trajectory inference analysis of integrated control and experimental datasets. D, RNA velocity (gene transcription) and gene expression (mature mRNA level) in clusters. E-F, Integration of control and experimental datasets (Sympathetic overdrive - Nor) with cells of stromal vascular fraction of obese patient. Cluster 5 of contractile cells is integrated with smooth muscle cells from stromal vascular fraction (F). Nor – noradrenaline.

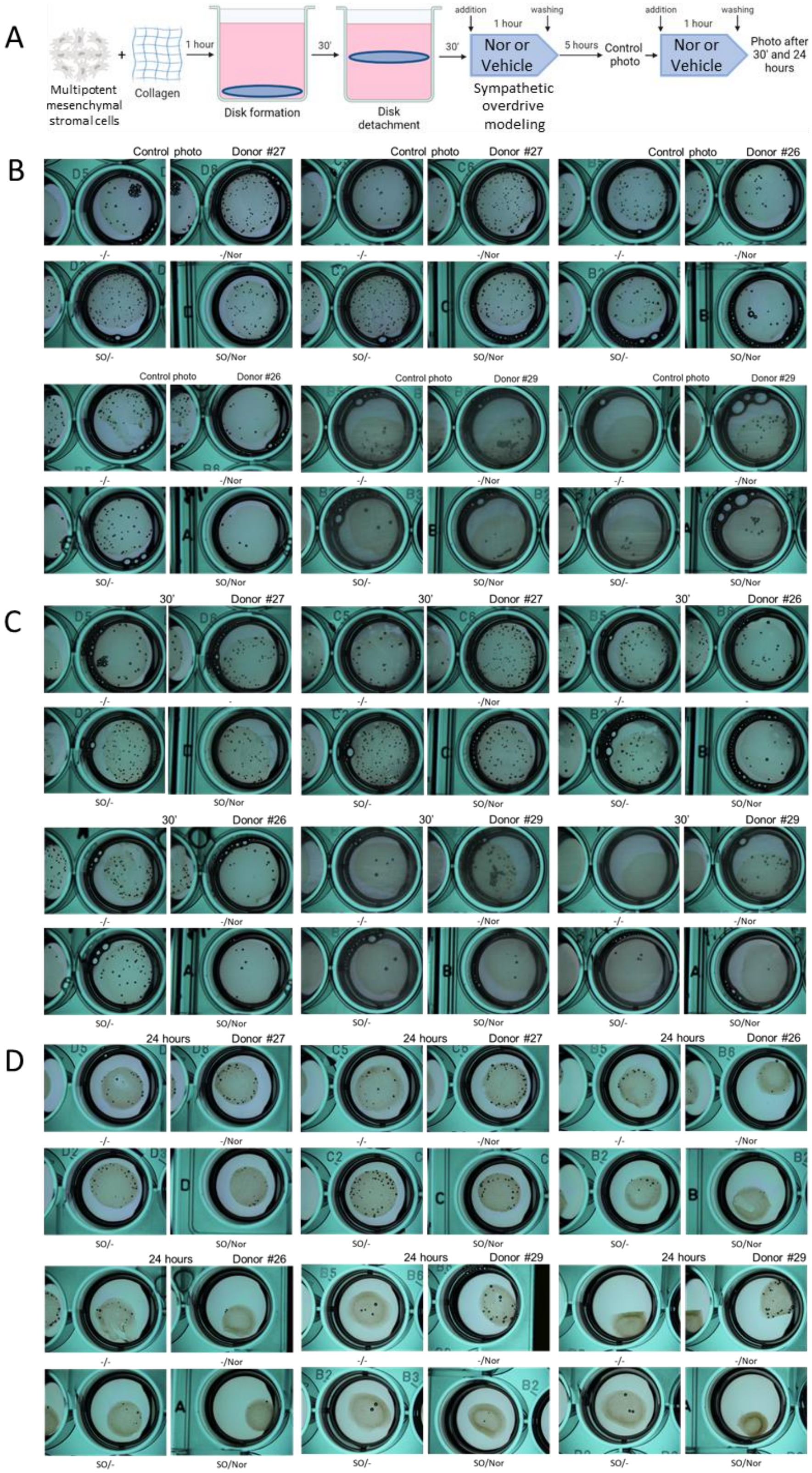

Figure S5. Collagen disks with MSCs contraction before, 30 minutes, 24 hrs after 2nd noradrenaline addition. A, scheme of experiment measuring contraction of collagen disks with MSCs. B, representative images of disks with MSCs before 2nd noradrenaline addition of 3 donors in 2 repetitions. C, representative images of disks with MSCs 30 minutes after 2nd noradrenaline addition of 3 donors in 2 repetitions. D, representative images of disks with MSCs 24 hours after 2nd noradrenaline addition of 2 donors in 2 repetitions. Nor – Noradrenaline (1 µM), SO – sympathetic overdrive.

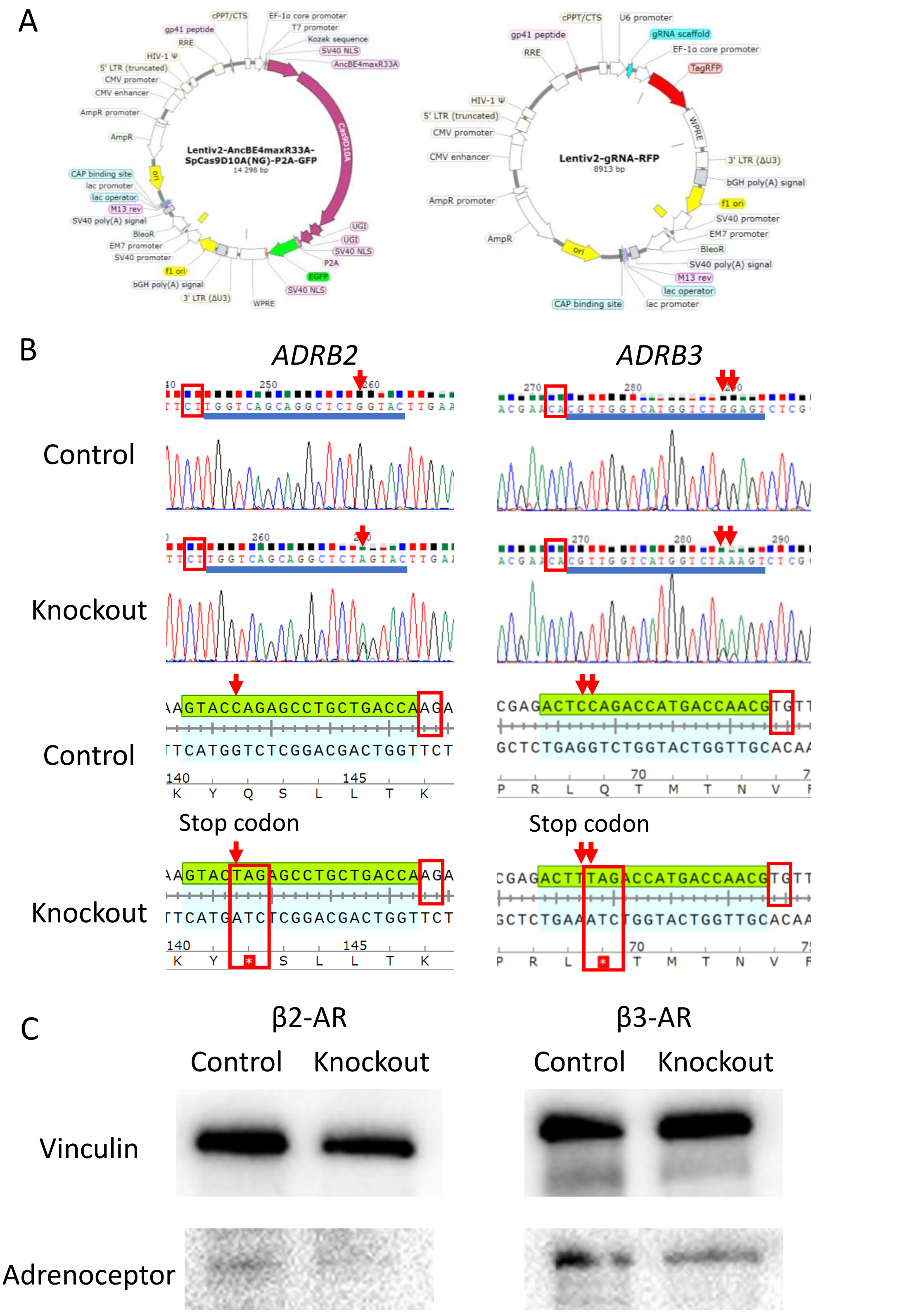

Figure S6. Confirmation of CRISPR/Cas9-mediated knockout of *ADRB2/ADRB3* genes in ASC52telo cell line. A, Lentivirus constructs used to knockdown *ADRB2/ADRB3* genes. B, amplicons of *ADRB2* or *ADRB3* knockout genomic DNA. C, western blot analysis of β2- and β3-adrenergic receptors after *ADRB2* or *ADRB3* knockout in ASC52telo. AR – adrenergic receptor.

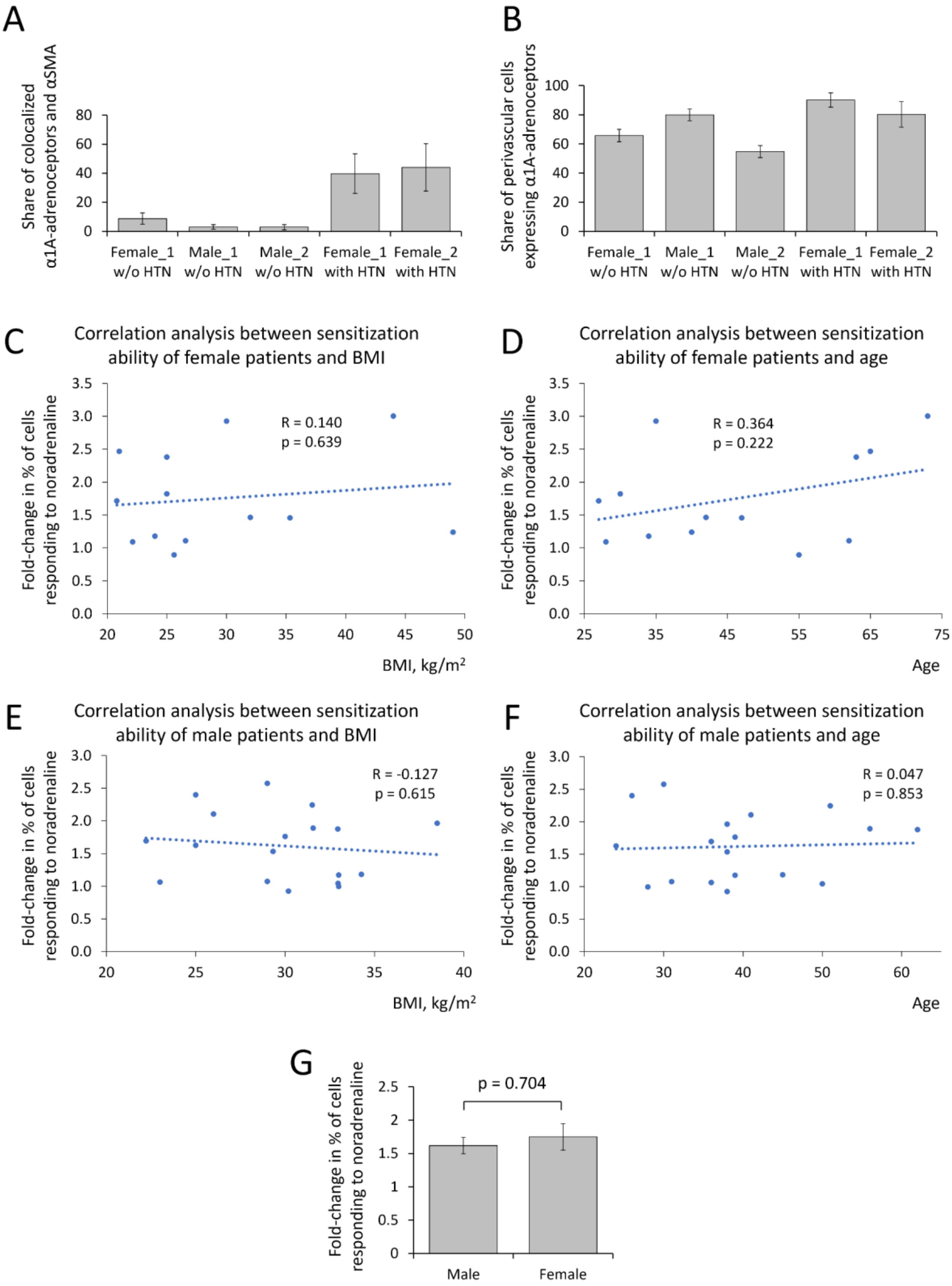

Figure S7. **Correlation analysis between BMI, age or sex and sensitization to noradrenaline ability of MSCs.** A, Share of perivascular cells with alpha1A-adrenoceptor colocalization with alpha-smooth muscle actin calculated based on immunofluorescent staining. B, Percent of perivascular cells expressing alpha1A-adrenoceptor in normotensive patients with obesity and hypertensive patients with obesity. C-D, Correlation between sensitization to noradrenaline after sympathetic overdrive modeling in female patients and BMI (C) or age (D). E-F, Correlation between sensitization to noradrenaline after sympathetic overdrive modeling in male patients and BMI (E) or age (F). G, Comparison between male and female noradrenaline-dependent sensitization ability. Mean ± SEM, n = 3-16.
