# Supplementary material for: Alpha1A- and Beta3-Adrenoceptors Interplay in Adipose Multipotent Mesenchymal Stromal Cells: A Novel Mechanism of Obesity-Driven Hypertension
Source: Cells. 2023 Feb 11;12(4):585. doi: 10.3390/cells12040585 (PMC9954306; doi:10.3390/cells12040585)
Supplement: Supplementary file 1 [file cells-12-00585-s001.zip › Supplemental Table S2. scRNAseq integrator clusters.pdf]

Cluster 0 of Integrated massives

Gene	Average fold-change	Average fold-change, log2	p-value	p_val_adj Adjusted p-value, based on bonferroni correction	pct.1 The percentage of cells where the feature is detected in the first group	pct.2 The percentage of cells where the feature is detected in the second group
HIST1H4C	3.335087	1.7377245	0	0	0.998	0.947
H2AFZ	2.206752	1.1419245	0	0	1	0.985
DEK	2.115831	1.0812244	0	0	0.996	0.91
TOP2A	2.00465	1.0033503	0	0	0.835	0.484
HMGB2	1.911566	0.9347553	0	0	0.925	0.653
HMGB1	1.831718	0.8731973	0	0	1	0.999
HMGN2	1.812739	0.8581709	0	0	0.999	0.948
STMN1	1.780906	0.8326112	0	0	0.959	0.744
PTMA	1.772437	0.8257341	0	0	1	0.999
RRM2	1.768526	0.8225476	0	0	0.874	0.478
MKI67	1.761884	0.8171191	0	0	0.784	0.365
CENPF	1.759838	0.8154424	0	0	0.751	0.365
CLSPN	1.736883	0.7965005	0	0	0.915	0.447
CKS2	1.736713	0.7963593	3.35E-263	6.97E-259	0.878	0.648
TYMS	1.716062	0.7791017	0	0	0.911	0.543
SMC4	1.715741	0.7788319	0	0	0.934	0.622
BIRC5	1.705406	0.7701153	0	0	0.863	0.446
PRC1	1.702334	0.767514	0	0	0.851	0.422
PCLAF	1.701986	0.767219	0	0	0.909	0.531
UBE2S	1.682761	0.7508305	3.54E-263	7.36E-259	0.968	0.849
TPX2	1.658932	0.7302548	0	0	0.818	0.441
ATAD2	1.655813	0.7275397	0	0	0.917	0.549
ANP32B	1.650677	0.7230579	0	0	0.992	0.895
UBE2C	1.63426	0.7086379	2.30E-275	4.79E-271	0.747	0.397
DTYMK	1.595867	0.6743402	0	0	0.914	0.633
CKS1B	1.586007	0.6653987	0	0	0.872	0.515
CEP55	1.576644	0.656857	0	0	0.748	0.302
TMPO	1.552625	0.6347096	0	0	0.854	0.475
H2AFX	1.54523	0.6278212	0	0	0.842	0.514
DUT	1.539556	0.6225146	0	0	0.96	0.793
HELLS	1.534683	0.6179406	0	0	0.825	0.376
RRM1	1.531234	0.6146952	0	0	0.912	0.584
PBK	1.521405	0.6054046	0	0	0.704	0.252
DNMT1	1.517819	0.602	0	0	0.95	0.768
RANBP1	1.516195	0.600455	0	0	0.991	0.923
MCM7	1.512988	0.597401	0	0	0.836	0.418
CENPW	1.50694	0.5916222	1.35E-275	2.81E-271	0.888	0.603
PCNA	1.50691	0.5915928	2.83E-292	5.89E-288	0.901	0.609
NUSAP1	1.495774	0.5808925	0	0	0.666	0.255
SHCBP1	1.495736	0.5808556	0	0	0.803	0.35
GTSE1	1.491898	0.5771486	0	0	0.693	0.271
HNRNPAB	1.490626	0.5759182	0	0	0.997	0.964
USP1	1.488254	0.5736205	0	0	0.84	0.46

GMNN	1.487801	0.5731816	0	0	0.837	0.44
ASPM	1.484807	0.5702756	2.37E-285	4.94E-281	0.663	0.271
CENPK	1.483546	0.5690494	0	0	0.79	0.35
TK1	1.476049	0.5617404	1.43E-295	2.97E-291	0.892	0.606
TUBA1B	1.475268	0.5609767	0	0	1	1
HIST1H1B	1.473705	0.5594474	0	0	0.542	0.134
PTTG1	1.471744	0.5575268	5.50E-209	1.15E-204	0.718	0.405
TUBB4B	1.471016	0.5568127	8.03E-263	1.67E-258	0.998	0.99
UBE2T	1.46838	0.5542259	0	0	0.776	0.355
NUCKS1	1.467725	0.5535814	2.05E-282	4.27E-278	0.995	0.958
ANLN	1.465807	0.5516956	5.57E-284	1.16E-279	0.719	0.333
H2AFV	1.454974	0.5409934	6.11E-249	1.27E-244	0.975	0.875
SPDL1	1.453036	0.5390701	4.87E-213	1.02E-208	0.874	0.674
RBMX	1.447617	0.5336799	1.39E-270	2.90E-266	0.968	0.862
SRSF7	1.445791	0.5318592	6.66E-252	1.39E-247	0.97	0.853
CBX5	1.44492	0.5309896	2.81E-264	5.86E-260	0.929	0.733
CDK1	1.443693	0.5297643	5.57E-298	1.16E-293	0.648	0.256
PARP1	1.439106	0.5251733	9.66E-274	2.01E-269	0.877	0.576
DHFR	1.435241	0.5212931	3.72E-307	7.74E-303	0.816	0.438
DNAJC9	1.434187	0.5202336	7.42E-274	1.55E-269	0.855	0.544
CDKN3	1.432101	0.5181329	6.69E-229	1.39E-224	0.679	0.341
NOP56	1.42915	0.5151571	1.04E-219	2.17E-215	0.934	0.799
NASP	1.427851	0.5138458	5.02E-255	1.05E-250	0.845	0.525
TACC3	1.423062	0.5089981	0	0	0.676	0.266
CBX1	1.421594	0.5075095	2.98E-221	6.20E-217	0.932	0.774
PA2G4	1.417196	0.5030388	6.31E-247	1.32E-242	0.985	0.927
RPL22L1	1.416281	0.5021078	1.82E-216	3.79E-212	0.996	0.968
CKAP2	1.415341	0.5011502	1.74E-206	3.62E-202	0.675	0.355
KIF23	1.412763	0.4985191	9.37E-273	1.95E-268	0.709	0.332
RAN	1.410012	0.4957079	0	0	1	0.995
MCM4	1.406581	0.4921923	2.07E-293	4.32E-289	0.808	0.423
CENPU	1.405639	0.4912266	0	0	0.693	0.248
KPNA2	1.403292	0.4888151	1.03E-163	2.15E-159	0.788	0.542
ORC6	1.398484	0.4838641	0	0	0.738	0.304
CBX3	1.39743	0.4827764	1.10E-273	2.29E-269	0.992	0.952
SIVA1	1.397037	0.4823704	1.69E-232	3.52E-228	0.971	0.861
RPA3	1.39503	0.4802959	1.70E-216	3.54E-212	0.893	0.654
CACYBP	1.394806	0.4800646	2.73E-210	5.68E-206	0.926	0.749
CCNA2	1.394741	0.4799971	8.00E-308	1.67E-303	0.656	0.246
ZWINT	1.393556	0.4787708	0	0	0.736	0.327
SLC25A5	1.390695	0.4758056	1.23E-215	2.56E-211	0.982	0.914
SNRPB	1.390526	0.4756305	5.16E-249	1.07E-244	0.987	0.927
DIAPH3	1.388895	0.4739373	2.85E-261	5.93E-257	0.785	0.426
CCNB1	1.387634	0.472627	3.72E-166	7.74E-162	0.537	0.251
MCM5	1.385075	0.4699641	9.05E-258	1.89E-253	0.767	0.402
NCL	1.384779	0.4696554	3.69E-167	7.68E-163	0.996	0.979
PPM1G	1.381566	0.4663043	4.55E-212	9.48E-208	0.961	0.845
NSD2	1.379894	0.4645572	3.56E-264	7.42E-260	0.737	0.37
SNRPG	1.379143	0.4637716	1.22E-227	2.55E-223	0.998	0.967
PGP	1.378845	0.4634605	9.18E-226	1.91E-221	0.889	0.647

RPL39L	1.372989	0.45732	2.79E-249	5.80E-245	0.76	0.409
SNRPD1	1.372783	0.4571031	2.96E-198	6.17E-194	0.988	0.915
MYBL2	1.372585	0.456896	1.38E-293	2.89E-289	0.66	0.267
KPNB1	1.372283	0.4565777	2.18E-254	4.55E-250	0.997	0.982
FBXO5	1.369314	0.4534535	1.17E-268	2.44E-264	0.658	0.28
FABP5	1.369249	0.4533848	4.29E-122	8.93E-118	0.961	0.884
SRSF3	1.367359	0.451392	2.75E-171	5.72E-167	0.976	0.896
HNRNPD	1.367206	0.4512303	2.46E-212	5.12E-208	0.98	0.896
MCM3	1.366548	0.4505363	1.28E-227	2.66E-223	0.735	0.39
MAD2L1	1.361599	0.445302	0	0	0.688	0.271
FEN1	1.359941	0.4435443	1.25E-287	2.60E-283	0.724	0.322
LSM5	1.359528	0.4431054	1.22E-184	2.54E-180	0.927	0.741
LMO4	1.358751	0.4422815	1.22E-106	2.54E-102	0.996	0.978
TFDP1	1.358168	0.4416616	2.55E-204	5.31E-200	0.927	0.739
SERBP1	1.3572	0.4406338	2.38E-246	4.96E-242	1	0.99
PRR11	1.35409	0.4373237	2.76E-269	5.76E-265	0.631	0.247
SMS	1.351993	0.4350881	3.14E-161	6.54E-157	0.974	0.911
SLBP	1.351411	0.4344669	2.15E-162	4.47E-158	0.841	0.628
SSRP1	1.347429	0.4302093	2.13E-192	4.43E-188	0.892	0.676
YWHAH	1.34733	0.430103	1.62E-194	3.38E-190	0.992	0.95
CKLF	1.345951	0.4286259	2.65E-178	5.53E-174	0.898	0.701
KIF20B	1.343984	0.4265159	3.62E-270	7.53E-266	0.633	0.244
NCAPG	1.343122	0.4255903	0	0	0.616	0.201
CDC6	1.341086	0.4234022	2.39E-248	4.99E-244	0.658	0.285
GINS2	1.337769	0.4198287	9.08E-308	1.89E-303	0.653	0.239
RAD51AP1	1.337258	0.4192779	0	0	0.643	0.223
H3F3B	1.337102	0.4191092	1.24E-225	2.59E-221	1	0.993
BRCA1	1.336531	0.4184933	5.59E-279	1.17E-274	0.655	0.257
CENPE	1.33648	0.4184378	5.88E-179	1.23E-174	0.476	0.187
LSM2	1.335129	0.4169795	1.05E-175	2.19E-171	0.879	0.664
CDC20	1.334596	0.4164036	2.04E-229	4.25E-225	0.557	0.214
POLE3	1.33453	0.4163321	3.99E-188	8.32E-184	0.857	0.623
TCF19	1.333382	0.4150903	2.02E-273	4.21E-269	0.693	0.303
HNRNPA2B	1.332915	0.4145849	1.36E-188	2.83E-184	0.999	0.993
CMC2	1.332209	0.4138203	1.45E-173	3.03E-169	0.909	0.706
CENPX	1.331505	0.4130583	2.89E-162	6.01E-158	0.946	0.801
LSM4	1.330841	0.4123378	1.92E-167	4.01E-163	0.956	0.825
UHRF1	1.329693	0.4110934	2.59E-201	5.40E-197	0.706	0.37
PTGES3	1.328587	0.4098923	1.45E-182	3.02E-178	0.979	0.91
BAZ1B	1.327656	0.408881	5.58E-177	1.16E-172	0.888	0.674
HSPD1	1.327318	0.4085144	3.99E-161	8.31E-157	0.989	0.943
SRRM1	1.325777	0.4068386	4.10E-179	8.54E-175	0.985	0.913
C12orf75	1.323148	0.4039743	2.18E-170	4.54E-166	1	0.988
ARHGAP11A	1.322629	0.4034083	4.66E-214	9.71E-210	0.577	0.244
NAP1L4	1.31854	0.3989408	5.36E-175	1.12E-170	0.868	0.664
RBM8A	1.31784	0.3981754	5.43E-166	1.13E-161	0.972	0.896
HNRNPA1	1.3169	0.3971455	3.44E-174	7.17E-170	0.999	0.983
HMGN1	1.315464	0.395572	1.93E-173	4.01E-169	0.994	0.945
LMNB2	1.315297	0.3953883	4.87E-190	1.02E-185	0.778	0.482
FBL	1.315278	0.3953673	6.18E-170	1.29E-165	0.871	0.663

PSMC3IP	1.314645	0.3946738	3.11E-279	6.48E-275	0.637	0.243
SMC3	1.312884	0.3927392	1.09E-169	2.26E-165	0.833	0.593
MRTO4	1.31154	0.391262	1.00E-165	2.09E-161	0.862	0.634
CSE1L	1.305543	0.3846497	5.32E-202	1.11E-197	0.788	0.47
LSM3	1.305339	0.3844242	3.41E-159	7.11E-155	0.971	0.879
EMP2	1.304691	0.3837077	1.40E-176	2.92E-172	0.776	0.487
HNRNPA3	1.303198	0.382056	5.62E-152	1.17E-147	0.99	0.95
SKA2	1.30274	0.3815487	7.25E-158	1.51E-153	0.861	0.647
CARHSP1	1.301349	0.3800077	2.07E-137	4.30E-133	0.911	0.757
PSIP1	1.300563	0.3791367	4.37E-194	9.11E-190	0.718	0.402
FOXM1	1.299463	0.3779157	4.55E-218	9.47E-214	0.675	0.334
ANP32E	1.297079	0.3752664	1.06E-140	2.20E-136	0.826	0.613
LSM8	1.29566	0.373687	6.89E-145	1.43E-140	0.9	0.74
HMGA1	1.294401	0.3722849	1.18E-97	2.46E-93	0.98	0.933
DBF4	1.294368	0.3722474	8.05E-213	1.68E-208	0.614	0.281
SGO2	1.293782	0.3715945	8.14E-217	1.70E-212	0.558	0.222
SUPT16H	1.293067	0.3707975	1.24E-146	2.57E-142	0.921	0.769
FUS	1.290577	0.3680162	6.87E-160	1.43E-155	0.981	0.939
C19orf48	1.290468	0.3678947	4.41E-216	9.19E-212	0.685	0.339
RBM17	1.290194	0.3675881	2.01E-154	4.19E-150	0.912	0.735
POP7	1.289553	0.3668706	3.16E-150	6.58E-146	0.873	0.669
TCOF1	1.288634	0.365843	2.03E-184	4.22E-180	0.732	0.432
BTG3	1.287787	0.3648935	2.16E-162	4.50E-158	0.844	0.604
SMC2	1.287747	0.3648487	5.35E-201	1.11E-196	0.702	0.369
MRPL17	1.287468	0.3645368	2.78E-146	5.78E-142	0.955	0.841
ARPC5L	1.287226	0.3642656	6.98E-143	1.46E-138	0.946	0.836
EIF1AX	1.287062	0.3640817	1.81E-171	3.78E-167	0.994	0.968
BRCA2	1.286483	0.3634324	6.07E-267	1.26E-262	0.61	0.22
CHAF1A	1.284714	0.3614468	2.59E-242	5.41E-238	0.625	0.252
PKMYT1	1.282262	0.358691	2.48E-256	5.16E-252	0.597	0.219
RDX	1.282221	0.3586451	1.32E-157	2.75E-153	0.983	0.918
RFC1	1.279423	0.3554936	1.04E-141	2.17E-137	0.86	0.658
MCM6	1.27914	0.3551745	4.00E-171	8.34E-167	0.692	0.391
CDT1	1.27885	0.3548475	1.11E-246	2.31E-242	0.601	0.231
KHSRP	1.27877	0.3547573	6.91E-138	1.44E-133	0.906	0.762
HNRNPF	1.278589	0.3545521	8.18E-130	1.70E-125	0.926	0.792
KIF11	1.278213	0.3541278	6.13E-232	1.28E-227	0.545	0.197
RFC3	1.278059	0.3539544	9.48E-270	1.97E-265	0.596	0.211
ALYREF	1.277992	0.3538791	5.28E-138	1.10E-133	0.849	0.652
EZH2	1.277766	0.3536239	3.44E-218	7.17E-214	0.582	0.236
PLK1	1.276205	0.3518601	1.13E-135	2.35E-131	0.41	0.169
HNRNPR	1.275654	0.3512367	4.05E-114	8.44E-110	0.926	0.813
SRSF2	1.275616	0.3511935	1.06E-129	2.22E-125	0.975	0.906
NPM1	1.275395	0.3509437	7.36E-157	1.53E-152	1	0.999
TOMM40	1.275276	0.350809	2.07E-140	4.31E-136	0.891	0.718
CDCA8	1.27388	0.3492295	1.77E-204	3.69E-200	0.506	0.191
TRIP13	1.273398	0.3486838	6.44E-225	1.34E-220	0.599	0.247
TUBB	1.272026	0.347128	1.02E-245	2.13E-241	1	1
MELK	1.271922	0.3470105	5.95E-228	1.24E-223	0.627	0.272
SMC1A	1.271741	0.3468044	2.48E-149	5.17E-145	0.813	0.578

RAD21	1.271618	0.3466658	4.74E-120	9.88E-116	0.895	0.759
ARL6IP1	1.271426	0.3464472	4.41E-56	9.18E-52	0.755	0.624
DLGAP5	1.26866	0.3433053	1.12E-205	2.33E-201	0.45	0.145
POLD3	1.268635	0.3432769	5.92E-237	1.23E-232	0.582	0.223
SUMO2	1.266314	0.3406353	1.67E-148	3.49E-144	0.997	0.968
NCAPD3	1.265331	0.3395146	9.05E-203	1.89E-198	0.679	0.337
HSPE1	1.265296	0.3394752	1.16E-134	2.42E-130	0.999	0.984
ZWILCH	1.264012	0.3380102	6.73E-199	1.40E-194	0.63	0.298
HSP90AA1	1.262786	0.3366096	2.91E-131	6.07E-127	0.999	0.999
ARHGAP18	1.261877	0.3355709	5.31E-97	1.11E-92	0.824	0.67
HNRNPM	1.26179	0.3354721	1.27E-129	2.65E-125	0.99	0.954
RBBP8	1.260547	0.3340495	1.06E-164	2.20E-160	0.661	0.369
AURKA	1.260469	0.3339601	7.32E-134	1.53E-129	0.46	0.207
MIS18BP1	1.260321	0.3337912	1.13E-149	2.36E-145	0.682	0.411
CCT6A	1.259822	0.3332193	7.47E-127	1.56E-122	0.979	0.921
CDCA4	1.259723	0.3331067	7.34E-189	1.53E-184	0.583	0.272
PAICS	1.259356	0.3326866	2.64E-136	5.50E-132	0.83	0.612
DTL	1.25908	0.3323705	2.32E-233	4.83E-229	0.552	0.199
MCM2	1.258591	0.3318098	1.75E-196	3.65E-192	0.588	0.256
HMGB3	1.258384	0.3315722	2.22E-134	4.63E-130	0.664	0.402
FAM111A	1.257539	0.3306027	5.86E-164	1.22E-159	0.624	0.33
RNPS1	1.256805	0.3297603	5.47E-126	1.14E-121	0.915	0.773
BUB3	1.256132	0.3289881	2.06E-123	4.29E-119	0.875	0.711
PRKDC	1.255575	0.3283479	1.40E-147	2.93E-143	0.983	0.944
GGCT	1.254413	0.3270125	1.13E-129	2.36E-125	0.788	0.563
ESCO2	1.252638	0.3249699	2.45E-269	5.10E-265	0.485	0.131
CKAP2L	1.2508	0.3228506	2.91E-246	6.07E-242	0.501	0.155
KNL1	1.250752	0.3227961	2.00E-237	4.17E-233	0.51	0.164
HNRNPUL1	1.249583	0.3214467	6.27E-115	1.31E-110	0.937	0.844
E2F7	1.248623	0.3203375	1.84E-178	3.84E-174	0.569	0.257
SRSF10	1.248595	0.3203058	1.97E-120	4.11E-116	0.923	0.806
CCDC34	1.24767	0.3192367	2.22E-156	4.63E-152	0.612	0.323
FANCI	1.247351	0.3188676	1.40E-216	2.91E-212	0.566	0.221
SPC25	1.24597	0.3172692	3.31E-266	6.90E-262	0.464	0.121
TOPBP1	1.245807	0.3170809	1.45E-164	3.02E-160	0.639	0.341
BARD1	1.245166	0.3163377	4.31E-224	8.98E-220	0.563	0.212
AKAP12	1.245006	0.3161521	6.38E-59	1.33E-54	0.975	0.939
SNRPE	1.244655	0.3157456	9.56E-119	1.99E-114	0.992	0.953
ASF1B	1.244484	0.3155482	1.29E-249	2.68E-245	0.535	0.177
CENPM	1.244462	0.3155224	6.12E-181	1.27E-176	0.58	0.267
AURKAIP1	1.243613	0.3145371	5.04E-125	1.05E-120	0.992	0.956
NUP62	1.242943	0.3137605	1.02E-130	2.12E-126	0.706	0.459
CENPQ	1.242651	0.3134208	3.34E-213	6.95E-209	0.561	0.223
AURKB	1.241821	0.3124567	1.50E-211	3.13E-207	0.499	0.175
HMMR	1.24066	0.3111074	9.47E-193	1.97E-188	0.439	0.144
CDK4	1.240303	0.3106921	7.98E-103	1.66E-98	0.877	0.707
TRA2B	1.240111	0.3104689	9.47E-112	1.97E-107	0.92	0.803
DEPDC1	1.240073	0.3104248	1.84E-195	3.83E-191	0.442	0.146
HNRNPDL	1.240002	0.3103419	4.55E-105	9.48E-101	0.986	0.95
CDCA5	1.239324	0.3095529	3.01E-242	6.28E-238	0.516	0.168

LDHB	1.238745	0.308879	7.66E-122	1.60E-117	0.994	0.975
MZT1	1.238359	0.3084293	8.87E-124	1.85E-119	0.682	0.44
SET	1.237999	0.3080098	3.06E-107	6.38E-103	1	0.984
SNRPF	1.237217	0.3070982	2.67E-103	5.56E-99	0.985	0.929
ECT2	1.236344	0.3060801	4.06E-166	8.45E-162	0.565	0.263
LMNB1	1.236262	0.3059841	5.37E-226	1.12E-221	0.501	0.167
HAT1	1.236154	0.3058582	5.23E-141	1.09E-136	0.715	0.445
RACGAP1	1.235925	0.3055913	1.53E-186	3.18E-182	0.531	0.221
AMD1	1.235672	0.3052959	5.67E-87	1.18E-82	0.934	0.833
TOMM5	1.234161	0.3035306	2.04E-114	4.25E-110	0.998	0.973
MCM10	1.234145	0.3035122	3.05E-225	6.35E-221	0.483	0.153
MAGOH	1.234053	0.3034044	9.59E-97	2.00E-92	0.842	0.681
PDAP1	1.233458	0.3027081	8.83E-113	1.84E-108	0.98	0.924
HNRNPC	1.232968	0.3021356	6.55E-89	1.36E-84	0.989	0.959
CENPN	1.232675	0.3017928	2.18E-168	4.54E-164	0.608	0.307
RBBP7	1.232332	0.3013907	1.94E-113	4.05E-109	0.815	0.61
TPRKB	1.232323	0.3013808	5.10E-122	1.06E-117	0.704	0.459
NCAPG2	1.232077	0.3010923	2.94E-204	6.13E-200	0.539	0.21
LSM6	1.231244	0.3001168	5.88E-109	1.23E-104	0.763	0.539
SNX5	1.230626	0.2993927	2.27E-110	4.74E-106	0.787	0.583
WDR34	1.229972	0.2986254	1.27E-141	2.65E-137	0.654	0.381
XRCC5	1.229628	0.298222	4.92E-108	1.03E-103	0.968	0.911
UBE2I	1.229374	0.2979244	3.18E-108	6.63E-104	0.936	0.818
EXOSC8	1.229005	0.2974913	3.78E-158	7.88E-154	0.593	0.298
HDGF	1.228694	0.2971252	5.62E-84	1.17E-79	0.876	0.755
SDF2L1	1.22753	0.295758	2.12E-75	4.42E-71	0.954	0.909
NUP85	1.226759	0.2948514	1.00E-154	2.08E-150	0.625	0.334
MRPL14	1.22653	0.2945831	6.59E-101	1.37E-96	0.957	0.865
DCTPP1	1.226439	0.2944751	9.03E-115	1.88E-110	0.711	0.481
PSMG1	1.226353	0.2943748	6.33E-106	1.32E-101	0.802	0.596
BZW1	1.226045	0.2940117	1.32E-104	2.75E-100	0.993	0.978
SMCHD1	1.225892	0.2938323	3.99E-112	8.31E-108	0.741	0.509
PTMS	1.225867	0.2938026	2.68E-107	5.58E-103	1	0.989
TEX30	1.225828	0.2937569	1.42E-134	2.96E-130	0.639	0.373
SRP9	1.225325	0.293164	5.05E-95	1.05E-90	0.919	0.778
EIF4EBP1	1.224928	0.292697	1.63E-70	3.40E-66	0.968	0.888
DRAP1	1.224676	0.2924003	4.98E-108	1.04E-103	0.997	0.988
HIST1H1C	1.224616	0.2923293	3.04E-128	6.33E-124	0.52	0.264
ERH	1.224051	0.2916637	8.25E-104	1.72E-99	0.985	0.921
MCMBP	1.223647	0.291188	2.18E-128	4.54E-124	0.702	0.439
ILF2	1.223564	0.2910891	9.52E-100	1.98E-95	0.925	0.806
HNRNPH3	1.223461	0.2909683	3.02E-86	6.30E-82	0.925	0.835
PDCD5	1.222772	0.290155	5.28E-105	1.10E-100	0.994	0.96
ILF3	1.222474	0.2898033	1.79E-93	3.73E-89	0.9	0.763
HSPB11	1.221512	0.2886685	2.48E-102	5.17E-98	0.801	0.605
NUDT1	1.221501	0.2886554	8.14E-95	1.70E-90	0.82	0.633
METAP2	1.221126	0.2882123	9.76E-104	2.03E-99	0.981	0.94
CIP2A	1.220891	0.2879339	3.78E-195	7.88E-191	0.504	0.189
POLR3K	1.220834	0.2878676	4.56E-111	9.49E-107	0.761	0.529
COMMD4	1.220307	0.2872438	8.47E-96	1.77E-91	0.838	0.659

TPM3	1.220039	0.2869272	2.39E-126	4.98E-122	1	0.994
C21orf58	1.219482	0.2862685	5.28E-218	1.10E-213	0.48	0.157
FOXC2	1.219348	0.2861095	2.76E-69	5.75E-65	0.601	0.422
BANF1	1.218454	0.2850518	3.37E-92	7.03E-88	0.963	0.889
H1FX	1.217498	0.2839193	2.63E-61	5.48E-57	0.71	0.558
CCT5	1.216437	0.282662	9.78E-91	2.04E-86	0.971	0.916
LRR1	1.215986	0.2821272	5.54E-174	1.15E-169	0.552	0.246
HJURP	1.215855	0.2819709	7.05E-184	1.47E-179	0.433	0.145
NUDC	1.215576	0.2816407	1.05E-85	2.20E-81	0.929	0.843
MRPS6	1.215415	0.2814495	1.53E-74	3.18E-70	0.86	0.729
PPP1R14B	1.215405	0.2814367	1.02E-132	2.12E-128	1	0.995
PAGR1	1.215366	0.2813909	1.77E-112	3.68E-108	0.697	0.466
SUZ12	1.215209	0.2812044	8.24E-110	1.72E-105	0.733	0.506
AKR1C1	1.215123	0.2811025	2.25E-17	4.68E-13	0.765	0.734
MT1M	1.214866	0.2807974	5.44E-30	1.13E-25	0.866	0.765
HSPA8	1.213841	0.2795797	1.09E-88	2.28E-84	0.998	0.996
CRIP1	1.213807	0.2795395	9.26E-07	0.0192864	0.622	0.607
HNRNPU	1.213711	0.2794248	3.42E-83	7.13E-79	0.977	0.939
FOSL1	1.213566	0.2792524	3.59E-75	7.48E-71	0.864	0.734
ATAD5	1.213227	0.2788499	3.08E-233	6.41E-229	0.483	0.146
C1QBP	1.213192	0.2788084	3.77E-82	7.86E-78	0.965	0.909
KIF2C	1.212649	0.2781619	8.38E-199	1.75E-194	0.451	0.146
TCEA1	1.212595	0.2780979	4.05E-83	8.45E-79	0.935	0.825
RFC2	1.212065	0.2774669	2.34E-183	4.87E-179	0.535	0.223
SKA3	1.211778	0.2771255	4.81E-206	1.00E-201	0.486	0.17
E2F1	1.211619	0.276936	4.39E-190	9.14E-186	0.502	0.189
H3F3A	1.211384	0.2766566	2.32E-98	4.84E-94	0.997	0.973
BRIX1	1.211101	0.2763194	6.02E-112	1.25E-107	0.703	0.466
NOLC1	1.210651	0.2757834	8.19E-88	1.71E-83	0.773	0.582
CMSS1	1.210112	0.2751408	1.87E-95	3.89E-91	0.694	0.481
MRPS34	1.210026	0.2750383	4.06E-85	8.45E-81	0.941	0.833
RFLNB	1.209006	0.2738218	2.29E-62	4.76E-58	0.947	0.874
NUF2	1.207682	0.2722404	2.40E-202	5.00E-198	0.429	0.129
RAD18	1.207396	0.2718995	1.27E-140	2.65E-136	0.613	0.328
RHEB	1.207374	0.2718732	8.85E-80	1.84E-75	0.946	0.871
RPA1	1.206638	0.2709934	8.09E-123	1.68E-118	0.666	0.412
RAD23A	1.206421	0.2707339	5.63E-73	1.17E-68	0.924	0.808
WBP11	1.206179	0.2704435	1.54E-106	3.21E-102	0.745	0.521
PPIA	1.20572	0.2698945	1.71E-182	3.56E-178	1	1
CDCA2	1.205674	0.2698394	2.68E-208	5.59E-204	0.414	0.118
UBA2	1.205119	0.2691758	8.01E-95	1.67E-90	0.809	0.616
NCAPD2	1.204502	0.2684371	2.91E-134	6.06E-130	0.514	0.252
WDR76	1.204262	0.2681493	2.16E-136	4.51E-132	0.52	0.243
JPT1	1.204108	0.2679645	9.21E-63	1.92E-58	0.987	0.953
TRIM28	1.204046	0.2678911	6.85E-90	1.43E-85	0.917	0.805
DSN1	1.203947	0.267772	2.11E-156	4.40E-152	0.536	0.247
MIS18A	1.20359	0.2673442	4.46E-187	9.30E-183	0.483	0.179
YWHAQ	1.203556	0.267303	7.12E-91	1.48E-86	0.981	0.93
PSMA7	1.203466	0.2671953	1.64E-116	3.42E-112	1	0.995
GLRX5	1.20216	0.2656283	1.69E-85	3.51E-81	0.88	0.726

LYAR	1.202006	0.2654438	1.24E-104	2.58E-100	0.636	0.402
U2SURP	1.201099	0.2643548	3.57E-86	7.45E-82	0.913	0.787
NUDT21	1.200025	0.2630646	3.58E-97	7.45E-93	0.719	0.503
HMGXB4	1.199481	0.2624108	7.20E-98	1.50E-93	0.709	0.489
NOP58	1.198643	0.2614024	1.72E-85	3.58E-81	0.776	0.582
YBX1	1.198622	0.2613771	4.93E-149	1.03E-144	1	0.999
VRK1	1.198281	0.2609664	4.44E-157	9.26E-153	0.527	0.237
RGS10	1.198237	0.2609136	5.30E-76	1.10E-71	0.835	0.671
RPS2	1.198037	0.260673	3.60E-125	7.51E-121	1	1
MYO19	1.197985	0.2606103	1.71E-124	3.56E-120	0.616	0.348
EIF5	1.197302	0.2597872	3.75E-74	7.80E-70	0.973	0.93
PNN	1.197262	0.2597384	1.10E-72	2.28E-68	0.889	0.769
NCAPH	1.196777	0.2591541	1.41E-219	2.94E-215	0.444	0.127
DKC1	1.196744	0.2591151	3.71E-77	7.72E-73	0.793	0.632
HAUS6	1.196723	0.2590892	9.57E-130	1.99E-125	0.613	0.34
SAE1	1.196616	0.2589599	1.57E-92	3.27E-88	0.814	0.638
TUBG1	1.195963	0.258173	1.12E-92	2.33E-88	0.752	0.549
GLO1	1.195539	0.2576615	9.75E-78	2.03E-73	0.853	0.702
TUBA1C	1.195429	0.2575287	2.89E-78	6.02E-74	0.998	0.994
GNPNAT1	1.195412	0.2575076	6.07E-71	1.27E-66	0.841	0.701
SAC3D1	1.195372	0.2574598	2.60E-111	5.42E-107	0.569	0.333
CNBP	1.195325	0.2574025	2.44E-77	5.09E-73	0.94	0.843
SAMD1	1.195143	0.2571833	3.02E-82	6.30E-78	0.751	0.56
DNAJA1	1.195107	0.2571404	1.80E-69	3.74E-65	0.931	0.839
EEF1E1	1.194365	0.2562442	4.62E-84	9.63E-80	0.816	0.632
SGO1	1.194256	0.2561116	3.68E-213	7.66E-209	0.419	0.12
PTBP1	1.194055	0.2558698	7.79E-78	1.62E-73	0.968	0.898
RPS20	1.193696	0.2554359	2.23E-105	4.65E-101	1	0.994
SNRPD3	1.193323	0.2549843	5.03E-73	1.05E-68	0.946	0.867
RBBP4	1.193241	0.2548852	1.12E-78	2.33E-74	0.817	0.664
PHF19	1.193191	0.2548246	1.16E-126	2.41E-122	0.439	0.197
DDX39A	1.193033	0.2546344	1.03E-87	2.15E-83	0.664	0.454
GPATCH4	1.192993	0.254586	2.01E-69	4.19E-65	0.828	0.684
ODC1	1.192119	0.2535288	1.21E-59	2.52E-55	0.858	0.722
KHDRBS1	1.191719	0.2530447	1.06E-74	2.20E-70	0.956	0.901
HDGFL3	1.191582	0.2528784	1.24E-84	2.58E-80	0.724	0.52
SUMO3	1.191325	0.252567	8.91E-70	1.86E-65	0.945	0.854
RFC4	1.1909	0.2520518	4.84E-168	1.01E-163	0.503	0.202
PRELID1	1.190886	0.2520358	1.14E-90	2.37E-86	0.996	0.974
BCL2L12	1.190641	0.2517386	8.85E-137	1.84E-132	0.566	0.288
SPATS2L	1.19012	0.2511075	1.85E-69	3.85E-65	0.975	0.901
OIP5-AS1	1.189529	0.2503901	4.92E-86	1.03E-81	0.76	0.57
SRSF4	1.189391	0.2502235	4.96E-71	1.03E-66	0.888	0.748
PSMD14	1.189254	0.2500564	8.82E-80	1.84E-75	0.959	0.877

Cluster 1 of Integrated massives

Gene	Average fold-change	Average fold-change, log2	p-value	p_val_adj<br>Adjusted p-value, based on bonferroni correction	pct.1<br>The percentage of cells where the feature is detected in the first group	pct.2<br>The percentage of cells where the feature is detected in the second group
CDKN1A	1.592044	0.6708799	3.38E-41	7.05E-37	0.969	0.932
GLRX	1.338253	0.4203508	5.93E-81	1.24E-76	0.913	0.8
FTL	1.332768	0.414426	1.78E-139	3.71E-135	1	1
NUPR1	1.302594	0.3813871	1.13E-70	2.35E-66	0.984	0.937
RPL27A	1.298343	0.3766719	2.03E-203	4.23E-199	1	1
FTH1	1.280021	0.356168	1.85E-81	3.86E-77	1	1
MIR100HG	1.23979	0.3100955	1.55E-59	3.24E-55	0.966	0.918
RPL23	1.230271	0.298976	4.75E-154	9.90E-150	1	1
PEG10	1.22694	0.2950646	8.14E-11	1.70E-06	0.761	0.809
GLS	1.225286	0.2931187	2.08E-44	4.34E-40	0.989	0.973
FGF7	1.225106	0.292907	9.61E-37	2.00E-32	0.95	0.925
RPL13A	1.224824	0.2925743	1.20E-105	2.50E-101	1	1
RPS27L	1.221054	0.2881266	9.94E-85	2.07E-80	1	1
CEBPD	1.217586	0.2840238	3.73E-50	7.76E-46	0.783	0.641
RPS11	1.214608	0.2804904	1.17E-127	2.44E-123	1	0.999
RPS8	1.214157	0.2799547	3.33E-128	6.94E-124	1	1
TPT1	1.214123	0.279914	3.14E-148	6.55E-144	1	1
CITED2	1.209673	0.2746172	1.34E-31	2.78E-27	0.977	0.953
RPL31	1.208158	0.2728091	3.18E-95	6.63E-91	1	0.986
ATP6V1G1	1.204857	0.2688622	1.76E-84	3.67E-80	0.99	0.959
SAT1	1.204428	0.2683482	1.25E-40	2.61E-36	0.954	0.901
MIR22HG	1.198355	0.2610556	1.78E-64	3.72E-60	0.825	0.669
FGF2	1.197884	0.2604886	7.88E-22	1.64E-17	0.945	0.918

Cluster 2 of Integrated massives

Gene	Average fold-change	Average fold-change, log2	p-value	p_val_adj<br>Adjusted p-value, based on bonferroni correction	pct.1<br>The percentage of cells where the feature is detected in the first group	pct.2<br>The percentage of cells where the feature is detected in the second group
LRRC75A	1.673363	0.7427504	1.83E-286	3.80E-282	0.901	0.524
ACTA2	1.310829	0.3904798	6.90E-171	1.44E-166	0.981	0.929
TPM2	1.278702	0.3546798	2.91E-169	6.07E-165	1	1
TIMP1	1.199775	0.2627635	8.91E-150	1.86E-145	1	1
CALR	1.20624	0.2705175	2.19E-148	4.57E-144	1	1
TAGLN	1.202176	0.2656476	1.22E-141	2.55E-137	1	1
CALD1	1.200433	0.2635552	2.47E-134	5.15E-130	0.999	1
C1orf56	1.319412	0.3998947	2.69E-113	5.60E-109	0.587	0.351
CTNNB1	1.26872	0.3433743	1.75E-96	3.64E-92	0.935	0.923

B4GALT1	1.220672	0.2876751	8.99E-80	1.87E-75	0.951	0.93
AC091607.2	1.242921	0.3137341	2.57E-73	5.36E-69	0.503	0.345
CDC42SE1	1.192749	0.2542906	1.00E-41	2.09E-37	0.701	0.67
CTSD	1.245984	0.3172853	1.46E-20	3.03E-16	0.889	0.928
FUCA1	1.397166	0.4825032	6.13E-19	1.28E-14	0.321	0.262
SOD2	1.303406	0.3822864	2.18E-13	4.55E-09	0.535	0.559

Cluster 3 of Integrated massives

Gene	Average fold-change	Average fold-change, log2	p-value	p_val_adj Adjusted p-value, based on bonferroni correction	pct.1 The percentage of cells where the feature is detected in the first group	pct.2 The percentage of cells where the feature is detected in the second group
LRRC75A	1.994551	0.9960642	0	0	0.981	0.553
C1orf56	1.535312	0.618532	0	0	0.89	0.326
SNHG29	1.450564	0.5366137	6.59E-277	1.37E-272	1	0.988
FTH1	1.390601	0.4757081	4.72E-302	9.83E-298	1	1
MT1X	1.365531	0.4494616	2.46E-163	5.12E-159	1	0.959
COX7A1	1.361342	0.4450294	1.43E-166	2.97E-162	0.998	0.96
ATP5MD	1.360535	0.4441741	9.36E-222	1.95E-217	1	0.994
SERF2	1.356597	0.4399921	2.09E-295	4.35E-291	1	1
RPS12	1.351758	0.4348368	0	0	1	1
CTNNB1	1.342229	0.4246304	1.94E-161	4.04E-157	0.995	0.914
RPL28	1.3401	0.4223409	4.82E-296	1.00E-291	1	1
CDC42SE1	1.339354	0.4215369	1.45E-165	3.01E-161	0.931	0.634
MIF	1.338285	0.4203856	1.68E-209	3.50E-205	1	0.999
RPS21	1.333133	0.4148212	2.44E-265	5.09E-261	1	1
RPL41	1.331266	0.4127984	0	0	1	1
RPS28	1.32903	0.4103736	2.33E-307	4.85E-303	1	1
COPS9	1.325925	0.4069996	5.58E-165	1.16E-160	1	0.974
MT1E	1.323453	0.4043068	7.43E-168	1.55E-163	1	0.996
B4GALT1	1.314441	0.3944495	1.12E-139	2.33E-135	0.995	0.925
MT2A	1.312125	0.3919056	2.64E-220	5.51E-216	1	1
ATP5F1E	1.31155	0.3912732	1.08E-232	2.25E-228	1	1
OST4	1.30742	0.3867228	3.04E-208	6.33E-204	1	0.998
WSB2	1.297553	0.3757939	7.72E-130	1.61E-125	0.99	0.88
RPS27	1.295914	0.3739699	4.05E-248	8.44E-244	1	1
SEC61G	1.29573	0.3737655	9.95E-204	2.07E-199	1	0.999
UBL5	1.294904	0.3728452	1.16E-169	2.42E-165	1	0.992
RPL35A	1.293182	0.3709256	9.49E-204	1.98E-199	1	1
TMSB10	1.293029	0.3707547	1.65E-290	3.43E-286	1	1
RPL36	1.291654	0.3692198	2.99E-227	6.23E-223	1	1
UQCR11	1.291577	0.3691338	1.05E-164	2.18E-160	1	0.991
ATOX1	1.28136	0.357676	1.74E-153	3.63E-149	1	0.989
RPL29	1.279951	0.3560888	1.82E-214	3.79E-210	1	1
TMA7	1.274647	0.3500973	1.97E-178	4.11E-174	1	0.998
RPLP1	1.272673	0.3478612	3.21E-271	6.68E-267	1	1
SH3BGRL3	1.264662	0.3387514	3.48E-196	7.24E-192	1	0.999

CYTOR	1.26315	0.3370259	9.36E-136	1.95E-131	0.999	0.993
COMMD6	1.259163	0.3324654	9.03E-112	1.88E-107	0.999	0.959
RPL39	1.25848	0.3316823	2.89E-212	6.02E-208	1	1
NDUFB1	1.256388	0.3292823	5.54E-105	1.15E-100	0.999	0.941
POLR2L	1.25574	0.3285375	5.52E-158	1.15E-153	1	0.999
CDKN1A	1.255166	0.3278778	2.53E-147	5.27E-143	1	0.931
FTL	1.255051	0.327746	9.62E-171	2.00E-166	1	1
RPS27L	1.247515	0.3190574	5.60E-155	1.17E-150	1	1
COX7A2	1.246117	0.3174397	1.83E-126	3.81E-122	1	0.993
RPL34	1.243794	0.314748	2.55E-172	5.30E-168	1	1
ATP5MG	1.243721	0.3146632	6.10E-112	1.27E-107	1	0.989
RPS15A	1.240144	0.3105082	1.41E-159	2.94E-155	1	0.999
RPS23	1.236858	0.3066794	2.60E-167	5.43E-163	1	1
ELOB	1.236837	0.3066554	5.11E-125	1.07E-120	1	0.995
RPLP2	1.232369	0.3014348	4.83E-171	1.01E-166	1	1
BRI3	1.231778	0.300742	4.71E-130	9.81E-126	1	0.997
RPL37	1.231636	0.3005755	1.49E-164	3.10E-160	1	1
COX7C	1.231158	0.3000161	2.61E-100	5.43E-96	1	0.982
NDUFAF8	1.229451	0.2980146	9.53E-95	1.99E-90	0.998	0.95
HNRNPH1	1.228924	0.2973959	7.16E-85	1.49E-80	0.985	0.883
TMEM258	1.225696	0.2936011	3.83E-95	7.99E-91	0.998	0.975
SEC61B	1.22142	0.2885589	1.36E-117	2.84E-113	1	0.999
COX7B	1.22031	0.2872483	1.05E-85	2.18E-81	0.995	0.956
UQCRQ	1.220301	0.287237	1.31E-103	2.73E-99	1	0.992
HINT1	1.21924	0.2859816	1.40E-103	2.91E-99	1	0.993
TOMM5	1.219077	0.2857887	2.23E-90	4.65E-86	1	0.976
UQCRH	1.21742	0.2838275	1.98E-100	4.12E-96	1	0.989
UQCR10	1.216702	0.2829764	7.02E-93	1.46E-88	1	0.981
RPL12	1.215894	0.2820177	1.74E-118	3.63E-114	1	0.999
C19orf53	1.214661	0.2805542	2.18E-85	4.55E-81	1	0.974
KCNMA1	1.214631	0.2805182	1.09E-107	2.27E-103	0.934	0.617
TOMM7	1.213622	0.2793186	1.21E-100	2.52E-96	1	0.992
COX6B1	1.210614	0.2757389	1.08E-87	2.25E-83	0.999	0.977
IL33	1.210247	0.2753018	1.37E-103	2.86E-99	0.958	0.674
RPL32	1.209616	0.2745495	3.47E-140	7.24E-136	1	1
DDAH1	1.209169	0.2740155	8.15E-77	1.70E-72	0.992	0.898
NDUFB2	1.208843	0.273627	7.06E-94	1.47E-89	0.999	0.993
NAA38	1.207149	0.2716041	5.02E-70	1.05E-65	0.991	0.911
S100A13	1.206636	0.270991	1.16E-93	2.42E-89	1	0.989
RPL30	1.206538	0.2708739	1.39E-135	2.90E-131	1	1
COX8A	1.20489	0.2689016	1.20E-86	2.49E-82	0.999	0.987
AXL	1.201666	0.2650356	8.38E-84	1.75E-79	0.994	0.933
C4orf3	1.201666	0.2650355	4.59E-79	9.57E-75	0.999	0.955
RPL37A	1.200183	0.2632546	3.56E-135	7.42E-131	1	1
KRT10	1.198188	0.2608541	9.77E-68	2.04E-63	1	0.945
ATP5ME	1.196898	0.2593005	4.28E-75	8.92E-71	0.999	0.979
NDUFA1	1.196001	0.2582186	6.44E-82	1.34E-77	1	0.984
SPATS2L	1.193761	0.2555144	3.92E-64	8.16E-60	0.989	0.909
NDUFA11	1.193115	0.2547333	4.15E-76	8.64E-72	0.999	0.98
C4orf48	1.19284	0.2544008	8.91E-66	1.86E-61	0.994	0.93

NDUFA4	1.192217	0.2536464	7.55E-105	1.57E-100	1	0.998
RTL8C	1.189985	0.250944	1.56E-71	3.24E-67	0.998	0.969
RWDD1	1.189944	0.2508933	1.23E-60	2.55E-56	0.992	0.904
MT1M	1.189849	0.2507786	1.34E-77	2.79E-73	0.972	0.761

Cluster 4 of Integrated massives

Gene	Average fold-change	Average fold-change, log2	p-value	p_val_adj Adjusted p-value, based on bonferroni correction	pct.1 The percentage of cells where the feature is detected in the first group	pct.2 The percentage of cells where the feature is detected in the second group
CD63	2.539408	1.3444922	0	0	1	1
TIMP1	2.500168	1.3220249	2.33E-236	4.86E-232	1	1
NEAT1	2.463897	1.3009419	2.03E-214	4.22E-210	0.997	1
HSPA5	2.330586	1.2206927	1.39E-229	2.90E-225	1	0.995
HSP90B1	2.297412	1.2000099	9.96E-287	2.07E-282	1	0.999
POSTN	2.281118	1.1897412	8.31E-45	1.73E-40	0.942	0.97
TGFBI	2.202791	1.1393329	5.61E-227	1.17E-222	1	0.999
APLP2	2.185843	1.12819	9.56E-253	1.99E-248	1	0.996
PPIB	2.153984	1.1070073	6.98E-278	1.45E-273	1	0.999
COL3A1	2.129824	1.0907342	5.22E-237	1.09E-232	1	1
VCAN	2.095742	1.0674612	9.27E-225	1.93E-220	1	0.993
SPARC	2.083727	1.0591661	1.80E-285	3.74E-281	1	1
MALAT1	2.080469	1.0569088	1.16E-167	2.41E-163	1	1
LOX	2.078514	1.0555522	2.50E-224	5.20E-220	1	0.999
IGFBP7	2.05584	1.0397281	2.78E-220	5.79E-216	1	0.999
FN1	2.053147	1.037837	3.87E-210	8.06E-206	1	1
DCN	2.042792	1.0305423	4.18E-164	8.70E-160	0.997	0.983
DKK1	2.042193	1.0301195	5.88E-82	1.22E-77	0.971	0.98
LUM	2.021716	1.0155802	4.04E-133	8.42E-129	0.997	0.994
PTX3	2.01383	1.0099416	7.35E-09	0.0001531	0.797	0.851
P4HA2	1.98684	0.990476	2.37E-194	4.94E-190	0.98	0.941
COL5A1	1.969157	0.9775783	1.48E-224	3.08E-220	1	0.998
FBN1	1.967207	0.9761486	5.82E-205	1.21E-200	0.997	0.988
MFAP5	1.967088	0.9760618	1.50E-178	3.13E-174	0.997	0.981
COL6A1	1.966741	0.9758067	6.91E-241	1.44E-236	1	1
COL6A2	1.961298	0.9718086	3.74E-242	7.79E-238	1	1
LMAN1	1.958538	0.9697772	6.26E-215	1.31E-210	0.984	0.954
INHBA	1.942274	0.9577465	1.04E-75	2.17E-71	0.991	0.997
COL1A1	1.941922	0.9574856	8.03E-281	1.67E-276	1	1
CD248	1.93724	0.9540028	1.02E-184	2.14E-180	0.988	0.986
LRP1	1.936414	0.9533874	1.25E-252	2.61E-248	1	0.994
TIMP3	1.935803	0.9529322	7.03E-82	1.47E-77	1	1
CD9	1.935459	0.9526754	4.11E-125	8.55E-121	0.946	0.917
THBS1	1.928683	0.9476161	3.08E-63	6.41E-59	0.997	0.996
COL1A2	1.925095	0.9449295	1.02E-276	2.12E-272	1	1
IGFBP3	1.92465	0.9445964	1.77E-32	3.69E-28	0.991	0.993

LAPTM4A	1.921337	0.9421108	3.26E-236	6.79E-232	1	0.984
CEMIP	1.921117	0.9419455	2.98E-77	6.22E-73	0.972	0.972
PDIA3	1.912806	0.9356907	3.92E-236	8.16E-232	1	0.988
LOXL2	1.912548	0.9354961	5.56E-204	1.16E-199	0.999	0.996
MFGE8	1.90962	0.9332859	2.84E-121	5.93E-117	0.985	0.968
CTSL	1.90957	0.933248	1.66E-164	3.45E-160	0.983	0.962
SERPINE2	1.909463	0.9331669	3.88E-23	8.09E-19	0.906	0.926
COL4A1	1.909198	0.9329665	3.95E-113	8.23E-109	0.993	0.987
MRC2	1.901424	0.9270799	3.47E-189	7.24E-185	0.987	0.955
COL5A2	1.900127	0.9260961	2.95E-178	6.15E-174	0.996	0.99
THBS2	1.898105	0.9245596	1.02E-148	2.13E-144	0.994	0.991
CYBA	1.897803	0.9243302	1.16E-159	2.42E-155	0.984	0.969
SERPINH1	1.89081	0.9190047	1.36E-228	2.84E-224	1	0.999
ITGB1	1.884287	0.9140186	1.33E-298	2.77E-294	1	1
COMP	1.877941	0.9091517	2.67E-14	5.55E-10	0.855	0.897
CCN2	1.875332	0.9071458	1.51E-73	3.14E-69	0.997	0.996
COL6A3	1.871173	0.903943	1.88E-209	3.92E-205	1	1
SLC1A5	1.869825	0.9029035	1.97E-131	4.10E-127	0.978	0.956
CD81	1.867547	0.9011443	3.74E-227	7.80E-223	0.999	0.998
MEG3	1.866754	0.9005319	1.13E-119	2.36E-115	0.955	0.94
COL12A1	1.861086	0.8961447	2.15E-98	4.48E-94	0.956	0.951
IGFBP6	1.834648	0.8755036	3.05E-132	6.36E-128	0.996	0.991
CCDC80	1.834235	0.8751783	1.73E-100	3.61E-96	1	0.999
TMEM59	1.833112	0.874295	3.78E-160	7.87E-156	0.946	0.868
DST	1.828474	0.8706404	1.70E-148	3.53E-144	0.965	0.936
FBLN2	1.822435	0.8658672	1.17E-120	2.44E-116	0.969	0.946
LAMC1	1.820937	0.8646807	5.05E-164	1.05E-159	0.981	0.952
RRBP1	1.820357	0.8642217	3.78E-218	7.87E-214	1	0.997
PDIA6	1.817878	0.862255	1.57E-201	3.27E-197	0.993	0.987
TMED9	1.815464	0.8603384	1.10E-202	2.29E-198	0.997	0.987
TMBIM6	1.815428	0.8603094	1.80E-229	3.76E-225	1	0.997
STC2	1.813935	0.8591224	4.68E-64	9.76E-60	0.962	0.954
FLNA	1.811819	0.8574386	5.57E-249	1.16E-244	1	1
EMC7	1.811187	0.8569354	6.49E-159	1.35E-154	0.967	0.91
MGST1	1.809174	0.8553312	2.03E-181	4.24E-177	1	0.996
CALU	1.7974	0.8459115	8.85E-293	1.84E-288	1	1
B2M	1.791598	0.841247	1.77E-251	3.70E-247	1	1
HM13	1.785524	0.8363475	3.74E-174	7.79E-170	0.98	0.93
GREM1	1.780022	0.8318947	3.57E-10	7.43E-06	0.834	0.89
SERPINE1	1.779168	0.8312029	3.23E-181	6.74E-177	1	1
SLC3A2	1.775452	0.8281863	2.64E-117	5.50E-113	0.92	0.824
APP	1.764838	0.8195356	1.84E-144	3.84E-140	0.971	0.927
SPCS2	1.760643	0.8161026	1.96E-149	4.09E-145	0.969	0.935
UBC	1.751988	0.8089925	5.97E-193	1.24E-188	1	0.999
PLEC	1.748973	0.8065078	1.33E-164	2.76E-160	0.996	0.976
CD44	1.748553	0.8061616	1.45E-149	3.02E-145	0.999	0.991
HLA-C	1.745991	0.8040461	6.33E-155	1.32E-150	0.981	0.952
PRDX4	1.744943	0.8031803	1.17E-134	2.43E-130	0.951	0.923
ISLR	1.744903	0.8031472	2.14E-64	4.46E-60	0.956	0.95
FLNC	1.738054	0.797473	1.82E-104	3.79E-100	0.991	0.977

LTBP2	1.734057	0.7941512	3.00E-116	6.26E-112	0.987	0.977
P4HB	1.729359	0.7902377	4.57E-218	9.51E-214	1	0.998
CCN1	1.727946	0.7890581	1.63E-173	3.39E-169	0.999	0.998
HSPG2	1.726065	0.7874867	5.27E-140	1.10E-135	0.975	0.942
CALR	1.725765	0.7872358	1.10E-211	2.30E-207	1	1
CANX	1.723156	0.7850529	2.36E-188	4.92E-184	0.997	0.986
RCN3	1.716068	0.7791064	2.37E-143	4.94E-139	0.967	0.932
MYADM	1.715136	0.7783227	4.61E-126	9.61E-122	0.991	0.984
RABAC1	1.713028	0.7765484	8.21E-158	1.71E-153	0.999	0.995
DAD1	1.711094	0.7749186	1.61E-156	3.35E-152	0.994	0.996
CD59	1.708965	0.7731227	4.71E-204	9.81E-200	1	1
COPB1	1.708068	0.7723656	9.32E-158	1.94E-153	0.969	0.946
PLAUR	1.70596	0.7705842	1.02E-124	2.13E-120	0.961	0.939
FSTL1	1.704646	0.7694719	6.60E-187	1.38E-182	1	0.998
ITGA5	1.704003	0.7689281	2.07E-129	4.31E-125	0.996	0.987
AKAP9	1.703492	0.7684948	1.77E-130	3.68E-126	0.962	0.922
COL4A2	1.702816	0.7679228	1.35E-103	2.80E-99	0.996	0.994
ITM2B	1.702669	0.7677978	1.25E-155	2.60E-151	0.988	0.964
OXTR	1.701562	0.7668594	4.20E-86	8.74E-82	0.953	0.959
ITGA2	1.69723	0.7631823	7.22E-62	1.50E-57	0.868	0.854
ATP2B1	1.697002	0.7629881	8.93E-95	1.86E-90	0.983	0.98
MXRA8	1.694118	0.7605346	2.16E-180	4.50E-176	0.999	0.995
SSR2	1.692016	0.7587433	1.28E-158	2.68E-154	0.991	0.986
EMP1	1.688598	0.7558262	4.81E-131	1.00E-126	0.994	0.989
SEC62	1.688419	0.7556731	2.31E-147	4.81E-143	0.997	0.984
BGN	1.687879	0.7552115	3.04E-99	6.33E-95	0.988	0.985
EMP3	1.684047	0.7519324	4.57E-146	9.52E-142	0.996	0.997
EFEMP1	1.683446	0.7514176	6.88E-76	1.43E-71	0.927	0.891
CDH2	1.68033	0.7487445	9.01E-146	1.88E-141	1	0.991
PLOD2	1.678139	0.7468619	7.58E-68	1.58E-63	0.917	0.896
DYNC1H1	1.674883	0.7440607	8.41E-149	1.75E-144	0.968	0.943
HLA-A	1.674657	0.7438654	1.18E-134	2.45E-130	0.955	0.905
SLC38A2	1.6716	0.7412295	1.02E-101	2.12E-97	0.993	0.986
MYDGF	1.670004	0.7398519	5.75E-172	1.20E-167	1	0.997
AHNAK	1.665358	0.7358324	3.52E-174	7.33E-170	1	0.998
SEMA7A	1.665175	0.7356741	3.02E-106	6.29E-102	0.971	0.944
ATP1B3	1.661955	0.7328812	6.55E-148	1.36E-143	0.98	0.947
PRSS23	1.66057	0.7316786	8.99E-82	1.87E-77	0.983	0.985
SEC61A1	1.658613	0.7299774	1.29E-138	2.68E-134	0.985	0.967
SLC16A3	1.657813	0.7292809	5.56E-131	1.16E-126	0.969	0.948
COL16A1	1.655734	0.7274708	3.05E-99	6.36E-95	0.855	0.758
DEGS1	1.654503	0.7263981	7.45E-126	1.55E-121	0.92	0.868
ATP6AP2	1.654018	0.7259746	1.52E-141	3.18E-137	0.938	0.861
ENG	1.652006	0.7242192	8.40E-175	1.75E-170	0.999	0.999
LGALS3BP	1.651924	0.7241476	2.58E-108	5.37E-104	0.901	0.819
CTHRC1	1.647406	0.7201959	4.68E-87	9.75E-83	0.968	0.957
LRPAP1	1.646364	0.7192834	5.16E-111	1.08E-106	0.868	0.764
RPN1	1.642251	0.7156742	1.16E-123	2.43E-119	0.953	0.917
ATP1A1	1.637278	0.7112989	1.95E-117	4.07E-113	0.948	0.907
ADAMTS1	1.636838	0.7109115	3.23E-28	6.74E-24	0.856	0.894

ALCAM	1.635087	0.7093676	3.38E-84	7.04E-80	0.981	0.972
PPIC	1.630899	0.7056677	4.69E-119	9.77E-115	0.956	0.907
REEP5	1.628145	0.703229	1.68E-120	3.50E-116	0.965	0.936
LAMB2	1.628128	0.7032141	1.41E-118	2.94E-114	0.881	0.773
LAMP2	1.626538	0.7018047	6.57E-114	1.37E-109	0.897	0.844
TMEM50A	1.625853	0.7011972	5.40E-128	1.12E-123	0.983	0.971
MMP14	1.624144	0.6996795	2.32E-126	4.84E-122	0.988	0.99
MMP2	1.622417	0.6981444	4.99E-129	1.04E-124	0.994	0.991
MANF	1.619249	0.6953244	6.17E-89	1.29E-84	0.971	0.962
TMED10	1.619126	0.6952154	1.82E-134	3.79E-130	0.961	0.943
PXDN	1.618213	0.6944017	2.17E-128	4.53E-124	0.981	0.966
TSPAN4	1.614737	0.6912992	2.67E-117	5.57E-113	0.962	0.933
DNAJC3	1.612579	0.6893701	9.93E-92	2.07E-87	0.875	0.846
SPOCK1	1.609037	0.6861972	6.54E-101	1.36E-96	0.949	0.93
CDH11	1.606459	0.6838844	6.55E-114	1.37E-109	0.971	0.95
SFRP2	1.606199	0.6836505	2.06E-07	0.0042922	0.497	0.503
PRKDC	1.604679	0.6822847	6.98E-102	1.45E-97	0.978	0.952
MEG8	1.603662	0.6813705	2.15E-71	4.48E-67	0.785	0.716
IQGAP1	1.599602	0.6777128	5.77E-153	1.20E-148	0.99	0.98
TIMP2	1.599391	0.6775225	1.54E-172	3.21E-168	0.999	0.999
OSTC	1.597754	0.6760453	6.74E-135	1.41E-130	0.996	0.993
HSPA8	1.596504	0.674916	1.40E-111	2.93E-107	0.994	0.997
RER1	1.591565	0.6704463	5.26E-127	1.10E-122	0.985	0.979
F3	1.589668	0.6687251	8.18E-22	1.70E-17	0.766	0.785
ERP29	1.58763	0.6668744	2.94E-107	6.12E-103	0.938	0.884
VMP1	1.586689	0.6660198	5.73E-90	1.19E-85	0.927	0.889
PDIA4	1.58496	0.6644465	1.93E-90	4.02E-86	0.955	0.915
SSR3	1.58003	0.6599523	3.05E-202	6.36E-198	1	0.999
CLTC	1.57564	0.6559376	2.68E-154	5.58E-150	0.991	0.987
ANO10	1.575344	0.6556672	1.18E-96	2.45E-92	0.868	0.808
FKBP10	1.573628	0.6540949	1.79E-104	3.73E-100	0.946	0.926
ADAMTS2	1.570781	0.6514822	2.46E-112	5.13E-108	0.968	0.944
SSR4	1.569106	0.6499431	7.63E-112	1.59E-107	0.977	0.978
FAT1	1.566058	0.6471376	5.13E-85	1.07E-80	0.85	0.778
RPN2	1.566022	0.6471043	2.98E-125	6.21E-121	0.975	0.956
CLU	1.565854	0.64695	1.87E-05	0.3903639	0.629	0.686
TMEM208	1.564653	0.6458429	2.86E-101	5.96E-97	0.953	0.918
ADAM12	1.563827	0.6450805	1.92E-109	3.99E-105	0.985	0.972
F2R	1.562752	0.6440888	2.72E-85	5.66E-81	0.983	0.974
GRN	1.561498	0.6429311	2.87E-108	5.99E-104	0.94	0.935
KDELR2	1.55927	0.6408709	6.52E-136	1.36E-131	1	0.996
PLOD3	1.558343	0.6400132	2.47E-99	5.14E-95	0.897	0.847
BCAP31	1.557907	0.6396095	1.03E-108	2.15E-104	0.953	0.9
SURF4	1.555112	0.6370181	3.30E-118	6.88E-114	0.993	0.974
DDX5	1.554733	0.636667	3.56E-127	7.41E-123	0.988	0.982
VASN	1.553261	0.6353002	3.46E-83	7.22E-79	0.942	0.936
CD55	1.551137	0.6333258	1.97E-65	4.10E-61	0.86	0.802
CD151	1.549616	0.6319108	1.50E-136	3.12E-132	0.997	0.988
NT5E	1.548385	0.6307642	2.71E-91	5.66E-87	0.975	0.969
SPON2	1.548147	0.6305428	6.66E-11	1.39E-06	0.622	0.668

SCUBE3	1.547176	0.6296372	7.97E-11	1.66E-06	0.818	0.89
P4HA1	1.546838	0.6293217	6.73E-84	1.40E-79	0.929	0.907
MCFD2	1.546603	0.6291027	3.55E-101	7.39E-97	0.985	0.972
DDOST	1.546514	0.6290201	1.12E-89	2.34E-85	0.804	0.672
GPC1	1.546253	0.6287762	1.21E-82	2.52E-78	0.901	0.858
LMO7	1.542804	0.6255545	3.75E-86	7.82E-82	0.988	0.988
HTRA1	1.542286	0.6250708	9.53E-86	1.99E-81	0.972	0.972
SCARB2	1.542017	0.6248183	6.85E-99	1.43E-94	0.942	0.9
LAMP1	1.540461	0.6233618	1.05E-118	2.19E-114	0.967	0.954
DCBLD2	1.539272	0.6222479	3.42E-92	7.13E-88	0.981	0.975
CTSC	1.537829	0.6208948	9.09E-42	1.89E-37	0.872	0.868
TFRC	1.53728	0.6203796	7.46E-97	1.55E-92	0.927	0.896
SYNE1	1.535359	0.6185757	1.33E-62	2.76E-58	0.862	0.818
BSG	1.533714	0.6170294	1.13E-102	2.36E-98	0.968	0.951
CKAP4	1.533316	0.6166555	1.10E-166	2.30E-162	0.999	0.998
DNAJB11	1.533035	0.6163903	5.86E-87	1.22E-82	0.865	0.79
THY1	1.532399	0.6157921	1.26E-83	2.63E-79	0.971	0.978
KCNQ1OT1	1.531433	0.6148823	7.74E-56	1.61E-51	0.664	0.532
FXYD5	1.530347	0.613859	9.78E-111	2.04E-106	0.971	0.961
MESD	1.529601	0.6131552	2.57E-97	5.36E-93	0.911	0.839
CERCAM	1.527903	0.6115525	1.11E-96	2.31E-92	0.911	0.844
FAP	1.525825	0.6095892	2.41E-83	5.03E-79	0.795	0.679
CRIM1	1.524073	0.6079323	3.04E-82	6.34E-78	0.994	0.993
ANPEP	1.522978	0.6068955	5.30E-44	1.10E-39	0.866	0.858
SLC39A7	1.522182	0.6061407	4.86E-98	1.01E-93	0.92	0.855
NPC2	1.522082	0.606046	4.68E-90	9.76E-86	0.993	0.982
ERP44	1.521951	0.6059219	2.83E-81	5.90E-77	0.724	0.562
ANTXR2	1.521492	0.6054872	3.87E-93	8.07E-89	0.919	0.857
IKBIP	1.520791	0.6048224	2.18E-92	4.53E-88	0.946	0.929
ATP2B4	1.520459	0.6045065	7.69E-70	1.60E-65	0.913	0.909
SIL1	1.519784	0.6038663	1.55E-84	3.23E-80	0.778	0.657
SELENOS	1.51854	0.6026854	2.56E-105	5.33E-101	0.987	0.984
HSP90AB1	1.518253	0.6024118	2.33E-133	4.86E-129	1	0.999
PDGFRA	1.51821	0.6023711	3.42E-47	7.13E-43	0.767	0.733
HAS2	1.518014	0.6021847	1.63E-22	3.39E-18	0.959	0.978
PSAP	1.516235	0.6004937	5.89E-110	1.23E-105	0.974	0.958
FLNB	1.515239	0.5995456	9.70E-65	2.02E-60	0.81	0.756
PIEZO1	1.515031	0.5993472	8.81E-85	1.84E-80	0.897	0.85
HEXB	1.514328	0.5986776	1.58E-84	3.29E-80	0.81	0.678
PTK7	1.514229	0.5985832	1.10E-57	2.28E-53	0.757	0.703
NRP1	1.512912	0.5973283	1.25E-83	2.61E-79	0.908	0.876
TGOLN2	1.512424	0.5968624	7.96E-105	1.66E-100	0.949	0.921
PRKCSH	1.510801	0.5953139	5.14E-88	1.07E-83	0.853	0.778
OLFML3	1.510761	0.5952758	8.26E-60	1.72E-55	0.778	0.702
EPRS	1.510214	0.5947533	9.30E-78	1.94E-73	0.898	0.88
MAGED2	1.509091	0.5936803	1.14E-85	2.37E-81	0.844	0.788
ITGAV	1.507126	0.5917998	7.67E-78	1.60E-73	0.906	0.875
P3H1	1.499929	0.5848942	1.71E-81	3.56E-77	0.788	0.672
RTN4	1.498664	0.5836769	6.66E-164	1.39E-159	1	1
MAGED1	1.494589	0.5797487	1.62E-84	3.37E-80	0.958	0.936

TXNDC15	1.494401	0.579567	6.89E-65	1.44E-60	0.714	0.594
HLA-E	1.49225	0.5774897	1.81E-89	3.77E-85	0.92	0.887
SSR1	1.490942	0.576224	1.40E-98	2.93E-94	0.964	0.951
TNC	1.490905	0.576188	4.80E-15	1.00E-10	0.657	0.684
PLAT	1.488572	0.5739289	2.48E-28	5.16E-24	0.667	0.626
TMCO1	1.488161	0.5735303	3.55E-84	7.40E-80	0.961	0.926
NID2	1.487402	0.5727946	6.46E-58	1.35E-53	0.772	0.692
CTSB	1.487048	0.5724509	1.38E-101	2.87E-97	0.993	0.994
ELN	1.486619	0.5720352	2.47E-13	5.14E-09	0.868	0.93
LMNA	1.485995	0.5714289	8.40E-123	1.75E-118	1	1
GANAB	1.485733	0.5711753	8.01E-95	1.67E-90	0.943	0.891
PCOLCE	1.48537	0.5708219	3.93E-52	8.18E-48	0.772	0.713
TMEM165	1.485266	0.5707211	8.42E-86	1.75E-81	0.919	0.882
SPTBN1	1.482129	0.5676714	8.55E-66	1.78E-61	0.856	0.832
COMT	1.480911	0.5664851	2.67E-86	5.56E-82	0.92	0.899
KDELR3	1.479159	0.5647773	2.05E-70	4.28E-66	0.888	0.89
PCDH18	1.478895	0.5645195	6.70E-36	1.40E-31	0.878	0.884
VCL	1.478532	0.5641655	3.45E-79	7.18E-75	0.971	0.962
CALD1	1.478455	0.5640908	5.90E-96	1.23E-91	1	1
ACTN1	1.477283	0.5629464	7.98E-120	1.66E-115	1	0.999
STT3A	1.475575	0.5612773	2.41E-90	5.02E-86	0.869	0.789
CLSTN1	1.474134	0.5598678	9.69E-75	2.02E-70	0.815	0.745
CLDN11	1.473614	0.5593591	7.29E-21	1.52E-16	0.812	0.843
SRPRB	1.471029	0.5568253	1.32E-82	2.75E-78	0.881	0.828
GLG1	1.469939	0.5557559	2.94E-78	6.12E-74	0.884	0.838
SLC4A7	1.469534	0.5553591	1.13E-56	2.34E-52	0.834	0.788
ASPH	1.469148	0.5549802	3.33E-87	6.94E-83	0.965	0.947
CTSA	1.468324	0.5541706	1.49E-83	3.11E-79	0.917	0.886
ATP2A2	1.467709	0.5535664	7.07E-78	1.47E-73	0.945	0.922
QSOX1	1.465332	0.5512277	5.89E-60	1.23E-55	0.824	0.809
SEC11A	1.462325	0.5482637	3.38E-87	7.05E-83	0.977	0.967
PLXNB2	1.461232	0.5471855	3.98E-60	8.29E-56	0.807	0.764
LAMA4	1.461137	0.5470917	3.12E-48	6.49E-44	0.794	0.761
PODXL	1.458789	0.5447708	2.21E-13	4.60E-09	0.782	0.822
MYH10	1.45734	0.5433378	9.44E-30	1.97E-25	0.847	0.866
CD164	1.454592	0.540615	1.10E-87	2.30E-83	0.94	0.902
ANTXR1	1.453428	0.5394598	8.93E-55	1.86E-50	0.846	0.851
CD99	1.452544	0.5385815	3.97E-132	8.26E-128	1	1
MYH9	1.451873	0.5379157	2.16E-106	4.51E-102	1	0.999
MACF1	1.451443	0.5374879	6.84E-73	1.43E-68	0.936	0.93
SDC2	1.450312	0.5363631	2.47E-60	5.14E-56	0.846	0.817
TM9SF2	1.450115	0.5361669	3.81E-68	7.94E-64	0.797	0.742
ATP6AP1	1.449941	0.5359942	2.97E-68	6.18E-64	0.85	0.789
LGMN	1.448628	0.5346869	1.51E-58	3.15E-54	0.827	0.797
EMILIN1	1.447067	0.5331322	1.58E-58	3.29E-54	0.782	0.715
HAS1	1.445962	0.5320295	5.49E-09	0.0001143	0.539	0.564
HERPUD1	1.445925	0.5319929	5.73E-49	1.19E-44	0.881	0.895
FKBP11	1.445588	0.5316569	1.26E-62	2.63E-58	0.907	0.892
SERINC3	1.445525	0.5315933	9.40E-67	1.96E-62	0.849	0.84
LAMB1	1.445351	0.5314201	3.97E-58	8.27E-54	0.751	0.663

LRP10	1.444562	0.5306324	8.23E-71	1.71E-66	0.939	0.93
PCYOX1	1.444204	0.5302742	5.95E-71	1.24E-66	0.839	0.772
GPNMB	1.443251	0.5293224	1.76E-63	3.66E-59	0.9	0.873
SPTAN1	1.4424	0.5284713	5.82E-81	1.21E-76	0.946	0.915
FSTL3	1.442246	0.5283174	1.94E-55	4.04E-51	0.939	0.926
PKD2	1.4403	0.5263689	1.45E-60	3.03E-56	0.837	0.785
TMEM41B	1.440265	0.5263339	1.32E-45	2.75E-41	0.823	0.807
HEG1	1.439382	0.5254496	4.87E-42	1.01E-37	0.795	0.759
CST3	1.438438	0.5245029	1.17E-90	2.45E-86	0.991	0.99
EIF4G1	1.437254	0.523315	1.00E-83	2.09E-79	0.969	0.97
OS9	1.436695	0.5227536	2.77E-68	5.77E-64	0.76	0.659
SULF1	1.435274	0.5213265	2.68E-23	5.58E-19	0.865	0.884
GLIPR1	1.43505	0.5211011	1.88E-47	3.91E-43	0.914	0.903
IGF2R	1.433737	0.5197799	2.42E-60	5.04E-56	0.817	0.755
TM9SF3	1.432204	0.5182367	1.31E-78	2.73E-74	0.953	0.935
PROCR	1.431783	0.5178125	8.21E-47	1.71E-42	0.693	0.616
SGCB	1.431625	0.5176536	6.09E-66	1.27E-61	0.83	0.754
CCDC47	1.431342	0.5173683	3.10E-59	6.46E-55	0.792	0.743
GOLGB1	1.430638	0.5166589	2.37E-58	4.93E-54	0.843	0.812
ARL6IP5	1.430438	0.516457	3.35E-69	6.97E-65	0.863	0.819
ECM1	1.428278	0.5142773	2.74E-42	5.70E-38	0.631	0.528
PGRMC2	1.426613	0.512594	2.41E-62	5.02E-58	0.879	0.833
DBN1	1.425408	0.5113747	3.01E-60	6.26E-56	0.891	0.884
FUCA2	1.424368	0.5103215	3.90E-65	8.13E-61	0.876	0.83
GSN	1.423149	0.5090872	6.18E-49	1.29E-44	0.807	0.79
SPCS3	1.42242	0.5083476	4.26E-71	8.88E-67	0.961	0.958
PAPPA	1.422407	0.5083339	2.15E-07	0.0044809	0.64	0.685
KDELR1	1.42116	0.5070694	8.85E-86	1.84E-81	0.984	0.973
SPTLC2	1.420889	0.5067939	5.50E-61	1.15E-56	0.773	0.699
ANXA1	1.419455	0.5053372	1.11E-91	2.31E-87	0.991	0.996
PLXND1	1.418922	0.5047956	3.75E-53	7.81E-49	0.859	0.848
VKORC1	1.418831	0.5047026	7.18E-85	1.50E-80	0.984	0.982
PMP22	1.417459	0.5033066	1.63E-61	3.39E-57	0.811	0.754
PTTG1IP	1.414278	0.5000662	3.56E-64	7.41E-60	0.92	0.902
AEBP1	1.411903	0.497641	1.87E-30	3.89E-26	0.804	0.814
ATP6V0E1	1.411547	0.4972767	2.33E-73	4.85E-69	0.999	1
SLC7A5	1.411435	0.4971624	1.21E-20	2.53E-16	0.701	0.708
SRPRA	1.411357	0.4970833	5.41E-59	1.13E-54	0.831	0.795
PLOD1	1.411251	0.4969748	2.86E-63	5.95E-59	0.855	0.813
NUCB2	1.410708	0.4964192	4.51E-66	9.40E-62	0.86	0.822
YIPF3	1.408825	0.4944924	5.06E-62	1.05E-57	0.831	0.802
SPPL2A	1.40873	0.4943946	8.80E-73	1.83E-68	0.808	0.721
TMEM30A	1.408315	0.4939698	7.36E-65	1.53E-60	0.866	0.82
CCPG1	1.407903	0.4935478	1.62E-51	3.38E-47	0.689	0.59
COPB2	1.407811	0.4934532	2.22E-73	4.62E-69	0.9	0.882
LMF2	1.4068	0.4924168	4.18E-54	8.72E-50	0.808	0.78
NUCB1	1.405912	0.4915067	8.77E-58	1.83E-53	0.828	0.776
MYOF	1.405094	0.4906668	1.53E-84	3.18E-80	0.987	0.987
SEL1L	1.404027	0.4895707	3.70E-65	7.71E-61	0.81	0.718
DERL1	1.403988	0.4895309	1.85E-60	3.86E-56	0.724	0.61

SLC52A2	1.403923	0.4894638	1.27E-55	2.66E-51	0.789	0.742
EMC3	1.402985	0.4884994	1.18E-49	2.45E-45	0.702	0.614
VCP	1.40179	0.48727	7.80E-74	1.62E-69	0.956	0.963
HEXA	1.401685	0.4871617	1.74E-52	3.63E-48	0.682	0.572
SDF4	1.400858	0.4863106	4.94E-70	1.03E-65	0.936	0.934
UTRN	1.397656	0.4830091	1.75E-50	3.66E-46	0.815	0.791
PSG5	1.397195	0.4825334	0.0002349	1	0.487	0.536
ORMDL2	1.396559	0.4818761	1.37E-51	2.85E-47	0.812	0.766
SLC8A1	1.396246	0.4815533	5.25E-38	1.09E-33	0.705	0.651
ACTN4	1.396064	0.4813653	3.26E-76	6.79E-72	0.999	0.995
CYP51A1	1.395171	0.4804417	1.85E-49	3.86E-45	0.779	0.738
ITGB5	1.394959	0.4802228	7.57E-71	1.58E-66	0.975	0.952
HLA-B	1.394373	0.479617	3.59E-29	7.47E-25	0.859	0.873
OCIAD1	1.393673	0.4788921	1.07E-55	2.22E-51	0.81	0.775
TMBIM4	1.393672	0.4788916	8.44E-54	1.76E-49	0.83	0.796
SCD	1.39307	0.4782675	4.02E-21	8.38E-17	0.769	0.784
MEGF6	1.392991	0.4781857	4.03E-35	8.40E-31	0.663	0.61
ATP6V0B	1.391913	0.4770688	6.16E-67	1.28E-62	0.981	0.979
DDR2	1.39152	0.4766621	3.66E-44	7.62E-40	0.839	0.831
IL6ST	1.388041	0.4730505	1.39E-57	2.89E-53	0.865	0.852
SON	1.387954	0.4729594	1.06E-67	2.21E-63	0.967	0.963
SF3B1	1.387624	0.4726167	7.59E-59	1.58E-54	0.865	0.844
NPTN	1.386723	0.4716799	2.87E-53	5.98E-49	0.858	0.85
PCMT1	1.386425	0.4713697	1.09E-45	2.27E-41	0.821	0.799
ADAM10	1.38498	0.4698649	2.10E-59	4.38E-55	0.812	0.742
TMED7	1.382173	0.4669381	4.78E-52	9.97E-48	0.849	0.824
MLEC	1.380988	0.4657005	8.38E-70	1.75E-65	0.972	0.959
PGRMC1	1.380217	0.4648952	5.19E-45	1.08E-40	0.862	0.854
IMPAD1	1.379411	0.4640528	4.04E-60	8.41E-56	0.855	0.819
RCN1	1.379126	0.4637543	1.01E-60	2.10E-56	0.945	0.932
FAM180A	1.37891	0.4635282	1.96E-31	4.08E-27	0.823	0.828
MAP1A	1.377079	0.4616113	1.19E-38	2.47E-34	0.853	0.855
EFEMP2	1.376455	0.4609569	1.89E-56	3.93E-52	0.923	0.914
LMAN2	1.376357	0.460855	2.41E-59	5.02E-55	0.903	0.876
DERL2	1.376252	0.4607445	1.98E-50	4.13E-46	0.757	0.71
KIDINS220	1.375128	0.4595661	4.76E-49	9.91E-45	0.773	0.707
AC020916.1	1.375099	0.459536	3.57E-37	7.44E-33	0.756	0.728
GLS	1.374952	0.4593811	2.38E-30	4.96E-26	0.968	0.978
PTPRK	1.374609	0.4590218	6.35E-40	1.32E-35	0.67	0.598
GOLGA2	1.373978	0.4583588	9.27E-61	1.93E-56	0.924	0.902
TRAM2	1.37362	0.4579834	7.35E-43	1.53E-38	0.932	0.924
NFE2L1	1.373126	0.4574644	3.32E-54	6.93E-50	0.887	0.871
STT3B	1.37287	0.4571949	5.51E-40	1.15E-35	0.847	0.85
CD276	1.371886	0.4561605	1.55E-45	3.23E-41	0.823	0.801
ITGA4	1.370023	0.4542001	1.01E-39	2.11E-35	0.693	0.622
TRAM1	1.37	0.4541762	1.40E-62	2.91E-58	0.94	0.949
ESYT1	1.36882	0.4529326	3.05E-48	6.35E-44	0.767	0.703
SERINC1	1.368436	0.4525275	2.67E-51	5.57E-47	0.86	0.848
EXT2	1.368137	0.4522126	1.15E-50	2.39E-46	0.75	0.693
GPX8	1.367007	0.4510202	1.46E-56	3.04E-52	0.943	0.944

NOTCH2	1.366533	0.4505206	6.16E-43	1.28E-38	0.772	0.752
FADS3	1.366429	0.4504101	4.13E-39	8.60E-35	0.677	0.601
GOLT1B	1.364972	0.4488714	5.15E-52	1.07E-47	0.818	0.783
LEPROT	1.364091	0.44794	4.11E-52	8.56E-48	0.876	0.868
YIPF5	1.363695	0.4475215	1.14E-59	2.38E-55	0.951	0.955
FOSB	1.362822	0.4465976	1.97E-16	4.10E-12	0.746	0.789
TNFRSF12A	1.362652	0.446417	1.61E-63	3.35E-59	0.997	0.997
MPZL1	1.362533	0.4462908	4.47E-51	9.32E-47	0.906	0.888
TOMM20	1.361624	0.4453278	2.39E-58	4.98E-54	0.961	0.973
NRP2	1.36117	0.4448468	2.48E-28	5.17E-24	0.756	0.743
ELOVL1	1.360324	0.4439506	2.85E-46	5.95E-42	0.826	0.804
CA12	1.360086	0.4436982	2.08E-26	4.34E-22	0.699	0.669
FKBP14	1.359537	0.4431159	7.89E-47	1.64E-42	0.765	0.7
SEC63	1.359284	0.4428474	6.29E-54	1.31E-49	0.894	0.873
CHPF	1.358956	0.4424989	4.41E-45	9.18E-41	0.891	0.884
TPR	1.357648	0.4411091	7.08E-43	1.47E-38	0.849	0.845
PRRC2C	1.356832	0.4402422	4.54E-62	9.46E-58	0.975	0.954
COLGALT1	1.356527	0.4399181	1.30E-45	2.70E-41	0.887	0.882
ANGPTL4	1.356287	0.439663	4.31E-14	8.97E-10	0.487	0.446
GPC4	1.35556	0.4388889	1.52E-26	3.16E-22	0.584	0.509
ALG5	1.355345	0.4386603	5.33E-46	1.11E-41	0.686	0.583
FUS	1.354909	0.4381957	1.53E-44	3.18E-40	0.943	0.951
GNS	1.354865	0.4381492	1.70E-49	3.55E-45	0.747	0.68
TLN1	1.352204	0.4353125	1.54E-70	3.21E-66	0.993	0.996
CAV1	1.351802	0.4348842	1.88E-32	3.91E-28	0.99	0.994
RTN3	1.351149	0.4341871	3.21E-49	6.69E-45	0.799	0.764
CRLF1	1.351023	0.4340522	6.02E-12	1.26E-07	0.828	0.89
MICA	1.350532	0.4335278	1.08E-39	2.24E-35	0.682	0.621
TMEM179B	1.350069	0.4330327	3.55E-42	7.40E-38	0.802	0.778
HSD17B12	1.349861	0.432811	2.73E-50	5.68E-46	0.86	0.837
GLB1	1.348883	0.4317656	2.12E-44	4.42E-40	0.634	0.532
ADGRE5	1.347905	0.4307185	3.45E-33	7.18E-29	0.751	0.747
ITGA11	1.3467	0.4294283	5.95E-24	1.24E-19	0.548	0.473
DNAJC1	1.346504	0.4292189	1.05E-38	2.19E-34	0.823	0.807
SPCS1	1.345943	0.4286176	2.37E-55	4.94E-51	0.985	0.971
ADAM9	1.345881	0.428551	1.64E-47	3.41E-43	0.855	0.834
BCAP29	1.345492	0.4281337	1.15E-37	2.40E-33	0.779	0.762
SQLE	1.344471	0.4270391	5.73E-23	1.19E-18	0.673	0.69
TPBG	1.34232	0.424729	8.30E-35	1.73E-30	0.754	0.741
RANBP2	1.342234	0.4246363	5.32E-45	1.11E-40	0.763	0.712
MBOAT7	1.342223	0.4246242	3.80E-44	7.91E-40	0.782	0.755
PDGFRB	1.342073	0.424463	6.25E-28	1.30E-23	0.74	0.731
MAN1B1	1.341855	0.4242286	2.18E-43	4.54E-39	0.66	0.573
CD47	1.341352	0.4236884	3.04E-35	6.34E-31	0.69	0.653
EMC10	1.340809	0.4231035	1.72E-45	3.58E-41	0.802	0.765
ERGIC3	1.340341	0.4226	8.21E-48	1.71E-43	0.843	0.833
EMC1	1.340212	0.4224614	2.57E-47	5.36E-43	0.693	0.613
FBLN5	1.338332	0.4204364	4.60E-35	9.58E-31	0.721	0.67
LIPA	1.337197	0.419212	3.63E-27	7.56E-23	0.689	0.653
FAF2	1.337126	0.4191351	6.12E-45	1.28E-40	0.706	0.623

TCIRG1	1.336059	0.4179835	1.84E-37	3.83E-33	0.581	0.469
EIF3A	1.335773	0.4176749	9.07E-59	1.89E-54	0.987	0.985
ITGBL1	1.335569	0.4174543	1.32E-13	2.75E-09	0.452	0.407
ACSL3	1.334759	0.4165796	1.83E-35	3.82E-31	0.673	0.62
CHPF2	1.334513	0.4163134	8.67E-38	1.81E-33	0.672	0.615
CTSD	1.334425	0.4162186	3.32E-51	6.92E-47	0.916	0.919
PLPP3	1.333561	0.4152839	3.16E-09	6.59E-05	0.603	0.635
MVP	1.333383	0.4150915	8.73E-49	1.82E-44	0.782	0.729
TXNDC5	1.332195	0.4138057	1.35E-53	2.81E-49	0.939	0.944
PLXDC2	1.331773	0.4133487	7.37E-34	1.53E-29	0.637	0.561
POLR2J3	1.331221	0.4127498	9.20E-37	1.92E-32	0.721	0.682
MAP1B	1.330338	0.4117928	2.42E-55	5.03E-51	0.999	0.998
SMPD1	1.330317	0.4117702	8.07E-39	1.68E-34	0.702	0.66
MANBA	1.329964	0.4113867	3.69E-41	7.69E-37	0.631	0.523
CREB3L2	1.32991	0.4113286	1.92E-31	4.01E-27	0.823	0.825
SLC35B2	1.329545	0.4109321	2.21E-40	4.60E-36	0.763	0.759
SLC16A1	1.329365	0.4107372	1.94E-32	4.03E-28	0.642	0.58
USO1	1.329226	0.4105869	1.84E-47	3.84E-43	0.843	0.833
GALNT2	1.328985	0.4103249	1.10E-40	2.30E-36	0.743	0.717
GBA	1.32872	0.4100369	2.63E-39	5.47E-35	0.705	0.649
FADS2	1.328322	0.4096045	4.09E-26	8.53E-22	0.695	0.693
LEPROTL1	1.32796	0.4092116	1.28E-42	2.66E-38	0.666	0.58
PIGT	1.326478	0.4076011	2.07E-41	4.32E-37	0.727	0.683
TMEM87A	1.326234	0.407335	9.69E-41	2.02E-36	0.735	0.675
HACD3	1.325775	0.406836	4.64E-36	9.67E-32	0.699	0.648
SEC31A	1.323331	0.4041745	3.65E-58	7.61E-54	0.971	0.979
HSP90AA1	1.323278	0.4041165	1.48E-70	3.08E-66	0.994	0.999
ZMPSTE24	1.323226	0.4040592	1.24E-39	2.58E-35	0.709	0.658
SLC20A1	1.323145	0.4039708	2.45E-19	5.10E-15	0.801	0.845
DIAPH1	1.322581	0.4033562	5.19E-35	1.08E-30	0.776	0.752
FKBP9	1.321991	0.4027124	6.29E-42	1.31E-37	0.818	0.793
SLC16A2	1.321697	0.4023919	1.12E-34	2.34E-30	0.577	0.486
TENM3	1.320674	0.4012739	8.10E-28	1.69E-23	0.757	0.759
LDLR	1.318814	0.3992406	1.91E-20	3.97E-16	0.599	0.572
BIRC6	1.317739	0.3980642	5.19E-35	1.08E-30	0.695	0.666
HUWE1	1.317302	0.397586	5.94E-36	1.24E-31	0.781	0.766
HNRNPU	1.316089	0.3962569	9.63E-38	2.01E-33	0.938	0.95
CPQ	1.315979	0.3961366	2.23E-34	4.65E-30	0.596	0.514
ATP6V0C	1.315834	0.3959774	3.14E-67	6.55E-63	0.994	0.997
XBP1	1.31577	0.3959078	6.53E-29	1.36E-24	0.856	0.873
DAG1	1.315084	0.3951544	1.77E-35	3.70E-31	0.698	0.644
KDM5B	1.314211	0.394197	1.67E-31	3.48E-27	0.708	0.687
XRCC5	1.31375	0.3936906	1.84E-42	3.84E-38	0.914	0.927
CLEC11A	1.31352	0.3934385	6.35E-34	1.32E-29	0.807	0.783
NCLN	1.311045	0.3907174	5.48E-34	1.14E-29	0.759	0.747
P3H3	1.30918	0.3886638	8.00E-30	1.67E-25	0.644	0.597
SLC31A1	1.30863	0.3880571	5.51E-30	1.15E-25	0.703	0.683
CTSK	1.308162	0.3875411	1.10E-17	2.29E-13	0.669	0.689
ADAMTSL1	1.307338	0.3866324	1.18E-30	2.45E-26	0.632	0.58
SLC7A8	1.306715	0.385945	9.68E-06	0.2016393	0.455	0.471

CLIC1	1.306041	0.3851998	2.74E-61	5.72E-57	0.996	0.998
TMX3	1.305181	0.3842498	3.32E-28	6.91E-24	0.612	0.568
DDRGK1	1.305039	0.3840929	4.48E-30	9.32E-26	0.618	0.545
ATP13A3	1.304887	0.383925	2.56E-33	5.34E-29	0.932	0.948
SLC39A14	1.304812	0.3838416	3.29E-34	6.86E-30	0.778	0.753
ERGIC2	1.304327	0.3833053	2.02E-36	4.20E-32	0.776	0.765
RNH1	1.304322	0.3833002	2.82E-48	5.87E-44	0.972	0.982
SPTLC1	1.304195	0.3831597	4.36E-33	9.09E-29	0.674	0.643
MFSD10	1.303704	0.3826161	2.32E-29	4.83E-25	0.677	0.658
KIRREL1	1.303575	0.3824731	6.27E-35	1.31E-30	0.703	0.657
NKTR	1.302986	0.3818221	6.87E-37	1.43E-32	0.749	0.707
UGGT1	1.301298	0.3799515	2.96E-33	6.16E-29	0.682	0.634
OSMR	1.29975	0.378234	3.12E-30	6.50E-26	0.645	0.6
P3H4	1.299531	0.3779906	4.09E-26	8.53E-22	0.77	0.774
SLC7A1	1.299458	0.3779095	4.56E-30	9.49E-26	0.844	0.844
FAM3C	1.299351	0.3777917	1.78E-38	3.71E-34	0.866	0.862
SCAMP3	1.297785	0.3760509	1.77E-31	3.69E-27	0.74	0.725
NID1	1.295996	0.3740617	3.61E-28	7.52E-24	0.69	0.657
TMEM214	1.295332	0.3733221	9.43E-28	1.97E-23	0.735	0.743
ADM	1.295071	0.3730317	1.17E-06	0.0243832	0.683	0.727
HYOU1	1.294654	0.3725669	3.04E-25	6.34E-21	0.651	0.621
TMEM131	1.294324	0.3721993	2.52E-25	5.25E-21	0.615	0.587
GLT8D1	1.293311	0.3710694	2.36E-29	4.91E-25	0.603	0.539
CARMN	1.292018	0.3696258	2.42E-18	5.03E-14	0.597	0.577
MXRA7	1.29113	0.3686339	1.10E-42	2.29E-38	0.977	0.978
CLPTM1	1.289426	0.3667286	2.02E-26	4.22E-22	0.727	0.727
CDH13	1.288665	0.3658777	9.24E-21	1.93E-16	0.658	0.652
SPNS1	1.288629	0.3658372	2.50E-35	5.20E-31	0.651	0.574
TSPAN3	1.288503	0.3656955	1.56E-30	3.25E-26	0.812	0.812
CBLB	1.288479	0.3656696	1.22E-21	2.55E-17	0.792	0.827
TMEM35B	1.286919	0.3639214	9.21E-31	1.92E-26	0.584	0.504
TECR	1.286535	0.3634908	2.61E-28	5.44E-24	0.663	0.622
CRTAP	1.286475	0.3634229	1.44E-36	3.00E-32	0.948	0.953
UGCG	1.285836	0.362707	1.12E-21	2.34E-17	0.597	0.557
PLXNA1	1.285629	0.3624741	9.01E-29	1.88E-24	0.605	0.553
GALNT10	1.285474	0.3623006	3.47E-26	7.22E-22	0.744	0.766
GOLIM4	1.285255	0.3620547	3.00E-29	6.25E-25	0.898	0.926
AKAP12	1.284432	0.3611308	1.62E-12	3.37E-08	0.908	0.952
PLP2	1.284384	0.3610763	1.09E-29	2.26E-25	0.924	0.944
SLC38A10	1.283791	0.3604107	3.97E-28	8.27E-24	0.647	0.615
SRGN	1.283476	0.3600567	4.03E-08	0.0008393	0.531	0.535
ANKLE2	1.283469	0.3600488	5.42E-31	1.13E-26	0.747	0.751
SYPL1	1.283458	0.3600366	7.16E-30	1.49E-25	0.715	0.708
FGFR1	1.283155	0.3596958	9.29E-33	1.93E-28	0.872	0.858
ATP13A1	1.282706	0.359191	2.54E-28	5.29E-24	0.547	0.449
MAN2B2	1.282493	0.3589506	1.39E-26	2.89E-22	0.542	0.463
DDX17	1.282296	0.3587292	1.34E-33	2.80E-29	0.852	0.877
CLMP	1.281864	0.3582435	8.41E-31	1.75E-26	0.913	0.916
LRRC32	1.281531	0.3578687	5.91E-18	1.23E-13	0.709	0.747
MSN	1.281165	0.3574563	5.55E-51	1.16E-46	0.997	0.995

ATF6	1.281084	0.3573645	1.59E-27	3.32E-23	0.632	0.586
ANO6	1.280481	0.3566856	1.53E-34	3.20E-30	0.757	0.754
SEMA3C	1.28042	0.3566171	2.38E-16	4.96E-12	0.589	0.574
MIRLET7BH	1.279396	0.355463	8.11E-19	1.69E-14	0.513	0.463
SCAF11	1.278853	0.354851	1.63E-34	3.40E-30	0.872	0.899
NECTIN2	1.278831	0.3548257	8.64E-28	1.80E-23	0.612	0.552
ELOVL5	1.278633	0.3546027	2.51E-24	5.23E-20	0.798	0.833
TPP1	1.277555	0.353385	4.40E-29	9.17E-25	0.64	0.581
CD109	1.277346	0.3531492	2.49E-26	5.19E-22	0.587	0.522
CD46	1.27726	0.3530527	1.23E-21	2.56E-17	0.577	0.551
ITGA3	1.277098	0.3528698	7.92E-12	1.65E-07	0.507	0.504
KRTCAP2	1.276855	0.3525948	1.53E-35	3.19E-31	0.981	0.98
POR	1.276514	0.3522092	4.24E-33	8.83E-29	0.583	0.483
PFKP	1.276466	0.352155	3.20E-33	6.66E-29	0.913	0.924
GLMP	1.276117	0.351761	2.40E-25	5.01E-21	0.701	0.725
PODN	1.276028	0.3516603	7.54E-24	1.57E-19	0.568	0.498
SLIT3	1.275603	0.3511795	1.55E-15	3.23E-11	0.578	0.571
SAR1B	1.275447	0.3510029	1.71E-29	3.56E-25	0.728	0.719
MAP4	1.274034	0.3494039	9.96E-39	2.08E-34	0.943	0.959
PCSK7	1.273766	0.3490997	1.33E-27	2.78E-23	0.802	0.823
N4BP2L2	1.273583	0.3488933	3.62E-25	7.55E-21	0.674	0.656
EDEM3	1.27339	0.3486738	1.18E-26	2.46E-22	0.609	0.562
CD36	1.273075	0.3483177	6.51E-06	0.135704	0.403	0.556
POFUT2	1.272704	0.3478974	3.94E-27	8.20E-23	0.6	0.543
B4GALT1	1.272439	0.3475968	1.31E-24	2.73E-20	0.929	0.936
ENPP2	1.272248	0.3473802	3.11E-09	6.47E-05	0.384	0.345
DHRS7	1.272071	0.3471794	3.89E-24	8.11E-20	0.637	0.624
UFL1	1.270952	0.34591	1.41E-27	2.94E-23	0.597	0.534
TRIP11	1.270788	0.3457231	1.12E-27	2.33E-23	0.728	0.718
MSMO1	1.270446	0.3453351	1.54E-15	3.22E-11	0.549	0.521
CHD9	1.269956	0.3447787	3.27E-29	6.81E-25	0.712	0.706
TUBA1A	1.269329	0.3440658	5.12E-27	1.07E-22	0.959	0.97
SLC2A3	1.269139	0.3438505	4.40E-22	9.17E-18	0.605	0.58
PAM	1.269129	0.3438392	4.52E-32	9.42E-28	0.811	0.818
XYLT1	1.269068	0.343769	9.86E-18	2.05E-13	0.603	0.589
TBL2	1.268866	0.3435393	1.15E-28	2.40E-24	0.578	0.506
SELENOF	1.268806	0.3434715	6.71E-35	1.40E-30	0.93	0.939
CCNL1	1.268443	0.3430589	1.95E-17	4.06E-13	0.594	0.593
NEU1	1.268308	0.3429051	2.95E-25	6.14E-21	0.584	0.541
NDFIP1	1.26819	0.3427706	1.73E-28	3.60E-24	0.741	0.733
STOM	1.268166	0.3427438	3.50E-21	7.30E-17	0.651	0.654
TKT	1.26799	0.3425439	8.49E-34	1.77E-29	0.884	0.888
VIM	1.267314	0.3417746	4.05E-86	8.45E-82	1	1
APMAP	1.267281	0.341737	1.51E-23	3.15E-19	0.661	0.638
LOXL1	1.267074	0.3415007	3.45E-25	7.18E-21	0.929	0.939
CCN3	1.26635	0.3406767	0.0042358	1	0.342	0.349
TM9SF4	1.266172	0.3404731	6.45E-26	1.34E-21	0.541	0.461
ERO1A	1.266064	0.3403502	2.61E-31	5.44E-27	0.731	0.704
ZNF638	1.266022	0.3403029	5.94E-25	1.24E-20	0.593	0.536
TUBA1C	1.266002	0.3402797	5.95E-35	1.24E-30	0.991	0.995

SPG7	1.265702	0.3399376	3.91E-22	8.15E-18	0.599	0.566
MXRA5	1.26455	0.3386237	1.71E-07	0.00356	0.404	0.382
TM9SF1	1.26439	0.338442	3.21E-24	6.69E-20	0.587	0.547
LIMA1	1.264265	0.3382993	4.91E-32	1.02E-27	0.987	0.989
MOGS	1.263989	0.337984	1.23E-19	2.57E-15	0.5	0.446
C1S	1.262991	0.3368444	2.71E-25	5.65E-21	0.52	0.434
COL11A1	1.262296	0.3360505	3.23E-08	0.0006725	0.419	0.391
BMP1	1.261993	0.3357038	4.12E-29	8.58E-25	0.663	0.622
SLC2A1	1.261647	0.3353081	3.79E-19	7.91E-15	0.526	0.456
ATXN2	1.261517	0.3351592	4.52E-27	9.41E-23	0.735	0.757
COL8A1	1.261438	0.3350688	3.36E-11	7.00E-07	0.452	0.419
PPP1R12A	1.26082	0.3343621	4.66E-35	9.70E-31	0.913	0.919
COPG1	1.260027	0.3334545	3.28E-31	6.83E-27	0.775	0.769
SDF2	1.259978	0.3333989	1.09E-27	2.26E-23	0.686	0.671
ITM2C	1.259899	0.3333076	1.27E-19	2.64E-15	0.618	0.607
PCDH9	1.259805	0.3332005	0.004321	1	0.403	0.435
ATRX	1.259075	0.3323638	8.88E-24	1.85E-19	0.781	0.796
ECE1	1.259043	0.332327	1.83E-25	3.80E-21	0.647	0.611
SACS	1.259011	0.3322904	1.05E-19	2.19E-15	0.724	0.742
MAGT1	1.25859	0.3318088	8.11E-27	1.69E-22	0.688	0.668
SETX	1.258302	0.3314787	3.17E-27	6.60E-23	0.693	0.666
PLBD2	1.258282	0.3314554	6.11E-26	1.27E-21	0.613	0.555
MBOAT2	1.257916	0.3310357	3.69E-20	7.69E-16	0.872	0.904
AXL	1.257716	0.3308062	1.71E-25	3.56E-21	0.938	0.942
ADAMTS6	1.25766	0.330742	1.09E-15	2.28E-11	0.632	0.647
GGH	1.257339	0.3303737	2.07E-17	4.32E-13	0.593	0.568
PON2	1.257299	0.3303277	2.47E-19	5.14E-15	0.66	0.674
M6PR	1.25723	0.3302483	4.84E-26	1.01E-21	0.718	0.721
MGAT1	1.256668	0.3296041	3.75E-24	7.82E-20	0.754	0.788
ABI3BP	1.256526	0.3294408	2.64E-10	5.50E-06	0.528	0.534
C1R	1.256494	0.3294041	1.28E-27	2.67E-23	0.458	0.341
KCNK6	1.25621	0.3290774	9.40E-20	1.96E-15	0.712	0.714
HSPA13	1.256068	0.3289147	1.55E-20	3.24E-16	0.58	0.545
PRNP	1.255905	0.3287268	2.88E-23	6.01E-19	0.717	0.719
SERPINB2	1.255839	0.3286516	0.0001216	1	0.224	0.33
RBM25	1.25581	0.3286181	1.87E-26	3.90E-22	0.833	0.848
TMEM43	1.255558	0.3283289	7.34E-25	1.53E-20	0.64	0.618
UBR5	1.255359	0.3281005	3.18E-24	6.63E-20	0.632	0.611
ADAM19	1.255163	0.3278743	5.24E-10	1.09E-05	0.674	0.731
ABL2	1.255102	0.3278042	1.16E-18	2.42E-14	0.804	0.846
BST1	1.254889	0.32756	1.86E-22	3.86E-18	0.587	0.549
SLC30A7	1.254335	0.3269231	3.11E-21	6.48E-17	0.599	0.581
DNASE2	1.253634	0.3261161	3.97E-24	8.26E-20	0.529	0.456
WSB1	1.253225	0.3256459	9.02E-23	1.88E-18	0.702	0.719
COPA	1.25317	0.3255824	3.44E-38	7.17E-34	0.948	0.95
DNAJB9	1.253017	0.3254054	2.43E-19	5.05E-15	0.584	0.549
SGCE	1.252777	0.3251293	6.35E-23	1.32E-18	0.542	0.48
FTX	1.252437	0.324738	2.35E-19	4.90E-15	0.471	0.391
USP9X	1.252037	0.3242772	2.87E-24	5.98E-20	0.859	0.891
TAPBP	1.251268	0.3233904	3.18E-27	6.62E-23	0.776	0.76

TMEM109	1.251019	0.323104	2.08E-20	4.32E-16	0.682	0.689
NAGLU	1.250767	0.322813	1.46E-23	3.04E-19	0.526	0.451
UBXN4	1.250179	0.322135	3.30E-27	6.88E-23	0.827	0.842
GOLM1	1.249978	0.3219031	2.97E-20	6.19E-16	0.621	0.603
BMPR2	1.249899	0.3218113	1.24E-24	2.57E-20	0.791	0.788
TM2D2	1.24986	0.3217661	1.40E-16	2.92E-12	0.603	0.618
AC092807.3	1.24984	0.3217436	3.34E-21	6.96E-17	0.706	0.72
DSE	1.249824	0.3217249	2.76E-23	5.76E-19	0.743	0.745
PTGES	1.2496	0.3214662	6.74E-10	1.40E-05	0.551	0.541
SLC38A1	1.24895	0.3207162	3.33E-06	0.0693842	0.557	0.594
SLC39A6	1.2488	0.3205424	1.39E-23	2.90E-19	0.731	0.743
PDIA5	1.248735	0.3204672	1.46E-26	3.04E-22	0.619	0.568
NCSTN	1.248523	0.320222	1.25E-20	2.61E-16	0.6	0.573
EGFR	1.247705	0.3192768	2.28E-14	4.76E-10	0.621	0.632
SLC35F5	1.246645	0.3180503	7.80E-22	1.63E-17	0.664	0.647
MARCH6	1.246535	0.317924	6.92E-22	1.44E-17	0.608	0.574
FAM234A	1.245653	0.3169027	5.36E-16	1.12E-11	0.616	0.63
IL7R	1.245618	0.3168618	1.15E-05	0.2386287	0.451	0.464
LENG8	1.244951	0.3160886	4.11E-21	8.57E-17	0.673	0.672
EEA1	1.244241	0.3152662	1.20E-23	2.49E-19	0.753	0.767
IL13RA1	1.244231	0.3152544	3.68E-22	7.67E-18	0.642	0.62
SLC4A2	1.244142	0.3151506	6.37E-22	1.33E-17	0.737	0.768
BOD1L1	1.243912	0.3148848	4.27E-18	8.90E-14	0.728	0.764
PGM3	1.243239	0.3141035	1.44E-26	3.00E-22	0.808	0.822
TUSC3	1.243192	0.314049	5.36E-22	1.12E-17	0.737	0.757
UNC5B	1.242794	0.3135874	8.22E-14	1.71E-09	0.597	0.574
WLS	1.24252	0.3132686	6.29E-17	1.31E-12	0.554	0.522
GARS	1.242215	0.3129151	8.58E-27	1.79E-22	0.936	0.957
NDUFA13	1.241972	0.3126321	1.09E-34	2.27E-30	0.987	0.996
UACA	1.241331	0.311888	4.22E-14	8.79E-10	0.82	0.867
DNAJC10	1.241257	0.3118024	7.52E-24	1.57E-19	0.749	0.75
CENPF	1.241138	0.3116638	6.70E-06	0.1396218	0.317	0.48
ASCC3	1.240191	0.3105623	7.78E-20	1.62E-15	0.722	0.751
TMEM200A	1.240076	0.3104282	6.50E-14	1.35E-09	0.547	0.527
C6orf89	1.239439	0.3096874	2.56E-26	5.33E-22	0.724	0.721
TMX2	1.239336	0.309567	1.59E-23	3.31E-19	0.65	0.625
PTGIS	1.239171	0.3093754	0.0005205	1	0.683	0.763
SND1	1.239053	0.3092375	4.12E-28	8.59E-24	0.826	0.83
ATL3	1.238686	0.3088108	2.50E-26	5.21E-22	0.855	0.862
IGFBP4	1.238548	0.3086499	7.28E-21	1.52E-16	0.996	0.997
KLC1	1.237729	0.3076955	2.06E-25	4.29E-21	0.83	0.86
TMEM259	1.237642	0.307594	3.29E-20	6.85E-16	0.648	0.666
HIF1A	1.237425	0.3073406	1.62E-34	3.38E-30	0.999	0.999
ASAH1	1.237162	0.307035	1.24E-19	2.57E-15	0.69	0.709
TMED3	1.236446	0.3061991	5.90E-20	1.23E-15	0.85	0.868
SNRNP200	1.23641	0.3061567	1.92E-21	4.00E-17	0.641	0.628
KDSR	1.236225	0.3059409	1.52E-23	3.17E-19	0.733	0.737
TMED5	1.235917	0.3055815	8.50E-18	1.77E-13	0.613	0.603
DNAJB4	1.235704	0.3053333	9.89E-11	2.06E-06	0.843	0.901
SH3PXD2A	1.235675	0.3052999	6.93E-22	1.44E-17	0.906	0.941

THOC2	1.234459	0.303879	6.05E-19	1.26E-14	0.69	0.712
VARS	1.234372	0.3037778	1.14E-18	2.38E-14	0.615	0.607
TMEM33	1.234238	0.3036204	2.94E-23	6.13E-19	0.65	0.627
FBLN1	1.23412	0.3034829	4.28E-13	8.93E-09	0.445	0.404
NEXN	1.233805	0.3031142	2.38E-22	4.95E-18	0.897	0.908
NDUFC2	1.233632	0.3029126	4.06E-26	8.45E-22	0.993	0.998
SLC38A5	1.233575	0.3028455	5.14E-11	1.07E-06	0.503	0.484
CNIH1	1.233392	0.3026317	5.58E-23	1.16E-18	0.794	0.822
EBP	1.232864	0.3020139	1.49E-15	3.11E-11	0.487	0.429
GPC6	1.232624	0.3017324	6.31E-16	1.32E-11	0.488	0.436
SAR1A	1.232097	0.3011163	1.13E-30	2.34E-26	0.894	0.911
FKBP7	1.232075	0.3010896	1.59E-24	3.32E-20	0.455	0.346
TMED4	1.231957	0.3009518	6.52E-22	1.36E-17	0.693	0.686
SRP54	1.231543	0.3004671	8.10E-22	1.69E-17	0.703	0.709
CLEC2B	1.231542	0.3004653	8.40E-06	0.1751043	0.411	0.404
ERLIN2	1.230998	0.2998289	5.23E-20	1.09E-15	0.49	0.421
EXT1	1.23077	0.2995612	2.17E-22	4.51E-18	0.932	0.96
UGGT2	1.230461	0.2991984	2.05E-25	4.28E-21	0.485	0.379
RIF1	1.229047	0.2975406	6.34E-15	1.32E-10	0.656	0.665
LNPEP	1.22896	0.2974382	2.32E-16	4.83E-12	0.535	0.507
TMEM87B	1.228911	0.2973801	1.06E-22	2.20E-18	0.571	0.514
TMED1	1.228708	0.2971425	4.50E-20	9.38E-16	0.558	0.516
PHTF2	1.227845	0.2961286	6.97E-15	1.45E-10	0.641	0.643
ITGA1	1.227791	0.2960652	5.72E-16	1.19E-11	0.517	0.469
CPD	1.227314	0.2955042	3.61E-16	7.51E-12	0.493	0.451
SMC1A	1.227275	0.2954583	2.35E-10	4.90E-06	0.619	0.642
PI16	1.22682	0.2949237	0.0001364	1	0.349	0.49
SDC1	1.226797	0.2948967	9.47E-08	0.0019732	0.539	0.558
HMGA2	1.226651	0.2947244	4.99E-14	1.04E-09	0.66	0.679
CCT3	1.225939	0.2938874	1.99E-26	4.15E-22	0.892	0.927
PLD3	1.225888	0.2938272	3.03E-19	6.32E-15	0.644	0.652
PRPF8	1.225501	0.2933718	2.82E-23	5.88E-19	0.735	0.748
PLA2G15	1.225197	0.2930137	3.05E-19	6.35E-15	0.468	0.396
PHGDH	1.225187	0.293002	1.33E-12	2.77E-08	0.624	0.637
SLC39A10	1.225044	0.2928338	8.82E-12	1.84E-07	0.51	0.493
NFASC	1.22503	0.2928174	1.16E-14	2.41E-10	0.519	0.498
TCTN3	1.224837	0.2925894	2.47E-18	5.14E-14	0.506	0.459
ATP1B1	1.224562	0.2922659	2.91E-14	6.06E-10	0.686	0.705
TNS1	1.224465	0.2921519	1.78E-23	3.71E-19	0.916	0.941
SLC39A13	1.224052	0.2916647	4.21E-20	8.77E-16	0.794	0.817
ACSL4	1.223939	0.2915316	9.27E-23	1.93E-18	0.83	0.862
ADAMTS4	1.223936	0.2915279	9.49E-11	1.98E-06	0.446	0.414
RDH11	1.22324	0.2907076	1.48E-11	3.08E-07	0.531	0.548
MALL	1.223219	0.2906831	2.64E-09	5.50E-05	0.913	0.928
PLXNA3	1.222975	0.2903952	5.31E-17	1.11E-12	0.51	0.472
ARL1	1.221562	0.2887276	6.33E-23	1.32E-18	0.805	0.826
SERPING1	1.221536	0.2886965	6.97E-18	1.45E-13	0.493	0.433
CEMIP2	1.221135	0.2882231	6.45E-18	1.34E-13	0.5	0.438
SLC38A7	1.22062	0.2876141	4.99E-17	1.04E-12	0.535	0.493
TXNDC12	1.220467	0.2874328	5.50E-15	1.15E-10	0.619	0.63

TOP2A	1.22042	0.2873779	3.76E-06	0.0784316	0.41	0.592
NOMO1	1.220321	0.2872601	1.53E-16	3.18E-12	0.522	0.493
B3GAT3	1.219946	0.2868177	3.38E-17	7.05E-13	0.667	0.688
TUFM	1.219647	0.286464	4.47E-22	9.32E-18	0.83	0.854
ATRAID	1.2195	0.2862898	5.55E-22	1.16E-17	0.801	0.807
SOAT1	1.218894	0.2855725	3.33E-13	6.95E-09	0.642	0.674
MIA3	1.21881	0.2854732	2.94E-14	6.12E-10	0.497	0.458
CNPY2	1.218567	0.2851852	9.33E-24	1.94E-19	0.935	0.948
WNT5B	1.21856	0.2851769	3.77E-10	7.86E-06	0.647	0.678
VPS13C	1.216923	0.2832383	3.62E-17	7.53E-13	0.513	0.468
MAN2A1	1.216918	0.2832324	8.37E-18	1.74E-13	0.657	0.657
AUP1	1.21681	0.2831037	3.62E-17	7.54E-13	0.744	0.78
HECTD1	1.216686	0.2829568	4.50E-18	9.37E-14	0.696	0.729
AKAP13	1.216303	0.2825024	6.19E-17	1.29E-12	0.599	0.584
LTBP3	1.216164	0.2823377	4.55E-17	9.49E-13	0.513	0.475
CHD4	1.21614	0.2823095	4.48E-21	9.33E-17	0.853	0.866
IFITM3	1.216076	0.2822331	3.35E-21	6.97E-17	0.996	0.995
TMEM181	1.215361	0.2813852	1.56E-17	3.25E-13	0.714	0.741
MFSD12	1.215085	0.2810574	3.20E-15	6.67E-11	0.66	0.669
ATP10A	1.214764	0.2806764	1.20E-12	2.51E-08	0.533	0.53
UXS1	1.214622	0.2805079	9.72E-17	2.02E-12	0.561	0.54
PTPN14	1.214571	0.2804471	4.59E-24	9.57E-20	0.884	0.902
PERP	1.214532	0.2804008	1.35E-09	2.82E-05	0.642	0.695
SLC33A1	1.214393	0.2802359	1.01E-14	2.10E-10	0.499	0.463
GNPTAB	1.214387	0.280228	6.71E-12	1.40E-07	0.503	0.495
LRP6	1.214049	0.2798262	5.24E-14	1.09E-09	0.519	0.485
ATP6V0A1	1.213567	0.2792539	1.82E-13	3.79E-09	0.484	0.456
CHST3	1.213557	0.2792423	1.08E-17	2.25E-13	0.574	0.554
ARL6IP1	1.213498	0.2791718	0.0001651	1	0.6	0.664
TOR1A	1.213279	0.2789115	2.01E-15	4.18E-11	0.586	0.584
DOCK7	1.213274	0.278906	9.14E-16	1.90E-11	0.561	0.559
CMTM6	1.213166	0.2787769	2.56E-15	5.34E-11	0.703	0.74
C16orf58	1.212888	0.2784459	6.06E-14	1.26E-09	0.507	0.488
NAV3	1.212549	0.2780429	2.63E-14	5.48E-10	0.702	0.74
SLC12A4	1.212535	0.2780258	3.40E-13	7.09E-09	0.544	0.538
THRAP3	1.212258	0.2776966	5.48E-18	1.14E-13	0.753	0.786
LARS	1.211509	0.2768054	6.62E-21	1.38E-16	0.753	0.773
UNC50	1.211007	0.2762067	8.21E-21	1.71E-16	0.513	0.438
HSPA1A	1.21041	0.2754963	1.88E-15	3.92E-11	0.574	0.544
EPG5	1.210125	0.2751562	1.72E-16	3.57E-12	0.612	0.606
ITFG1	1.210048	0.2750637	1.34E-16	2.79E-12	0.519	0.482
GFPT1	1.209977	0.27498	1.63E-21	3.41E-17	0.828	0.858
KIAA1549L	1.209703	0.2746526	1.25E-13	2.60E-09	0.568	0.566
SEC24D	1.209623	0.274558	2.35E-20	4.90E-16	0.799	0.853
ADAMTS7	1.209397	0.2742878	1.03E-17	2.15E-13	0.455	0.383
DYNC1I2	1.209273	0.2741406	5.45E-25	1.13E-20	0.956	0.956
FMOD	1.209086	0.2739166	8.25E-10	1.72E-05	0.459	0.433
SYVN1	1.209065	0.2738918	3.10E-13	6.46E-09	0.523	0.507
ATF6B	1.208768	0.2735379	6.21E-18	1.29E-13	0.512	0.463
PPT1	1.208425	0.2731282	5.92E-17	1.23E-12	0.626	0.619

PNN	1.208272	0.2729448	1.93E-14	4.01E-10	0.767	0.804
NEMF	1.208135	0.2727816	3.60E-19	7.50E-15	0.642	0.632
MYCBP2	1.207589	0.2721292	1.51E-15	3.15E-11	0.709	0.737
TTC14	1.207494	0.2720165	4.24E-13	8.83E-09	0.419	0.363
PLPP1	1.20748	0.2719997	2.14E-11	4.46E-07	0.564	0.576
APCDD1L	1.207431	0.2719409	0.0020539	1	0.593	0.672
PCNX1	1.207393	0.2718953	4.80E-16	9.99E-12	0.561	0.538
GM2A	1.207339	0.271831	3.63E-20	7.57E-16	0.517	0.457
LAPTM4B	1.206877	0.2712783	1.24E-13	2.58E-09	0.587	0.588
DHCR7	1.20682	0.2712108	6.81E-09	0.0001418	0.44	0.424
CTNNA1	1.206308	0.2705983	2.82E-25	5.87E-21	0.885	0.909
UBR4	1.206304	0.2705933	5.03E-17	1.05E-12	0.423	0.343
HGSNAT	1.206275	0.2705591	3.23E-14	6.74E-10	0.519	0.487
EHD2	1.20567	0.2698346	3.20E-24	6.66E-20	0.978	0.991
OGA	1.205281	0.2693692	1.01E-12	2.10E-08	0.544	0.54
HERC1	1.205243	0.269324	3.55E-20	7.39E-16	0.525	0.458
CYB561D2	1.205167	0.2692335	3.22E-15	6.70E-11	0.461	0.406
SCAMP2	1.204962	0.2689879	1.49E-16	3.11E-12	0.65	0.665
NBAS	1.20464	0.2686016	5.75E-19	1.20E-14	0.452	0.367
CNIH4	1.204563	0.2685098	1.73E-15	3.60E-11	0.781	0.824
SUN2	1.20409	0.2679434	3.99E-13	8.31E-09	0.501	0.477
ATP2C1	1.204077	0.2679276	4.44E-17	9.24E-13	0.622	0.619
TMEM47	1.204012	0.2678493	1.12E-13	2.34E-09	0.666	0.697
C1GALT1C1	1.203886	0.2676984	1.15E-24	2.39E-20	0.35	0.223
GIGYF2	1.203742	0.2675266	6.04E-15	1.26E-10	0.526	0.493
KIAA1109	1.203368	0.2670782	1.63E-18	3.39E-14	0.477	0.4
HSPA9	1.203337	0.2670413	2.43E-20	5.06E-16	0.904	0.935
IARS	1.203318	0.2670176	5.52E-19	1.15E-14	0.812	0.847
GTF3C1	1.203282	0.2669754	5.24E-16	1.09E-11	0.499	0.45
SLC17A5	1.203265	0.2669545	5.63E-13	1.17E-08	0.554	0.549
RNF149	1.201944	0.2653699	4.53E-13	9.45E-09	0.469	0.437
FLRT2	1.201889	0.2653031	0.0003639	1	0.465	0.505
JMJD1C	1.201503	0.2648399	1.90E-14	3.97E-10	0.536	0.508
CYB5R1	1.201339	0.2646433	1.13E-12	2.36E-08	0.494	0.461
YIF1A	1.201256	0.2645433	5.91E-16	1.23E-11	0.859	0.895
SUMF2	1.20123	0.2645129	4.54E-15	9.45E-11	0.66	0.686
SLC16A7	1.201135	0.264398	1.43E-16	2.98E-12	0.488	0.427
ILVBL	1.201035	0.2642783	3.50E-17	7.29E-13	0.43	0.349
TMEM115	1.200958	0.2641859	2.04E-11	4.26E-07	0.51	0.505
TMF1	1.200673	0.2638435	4.84E-19	1.01E-14	0.711	0.719
FAS	1.200293	0.2633867	4.49E-12	9.35E-08	0.577	0.576
SIRPA	1.199385	0.2622948	1.66E-12	3.47E-08	0.64	0.663
RCN2	1.199293	0.2621841	5.03E-20	1.05E-15	0.779	0.818
ATP8B1	1.199205	0.2620784	2.98E-06	0.0621355	0.528	0.579
PIGS	1.199193	0.2620637	1.57E-14	3.27E-10	0.526	0.503
RDH10	1.199179	0.2620466	0.0020074	1	0.44	0.468
PTPRF	1.198578	0.2613242	9.03E-10	1.88E-05	0.549	0.561
TMEM248	1.198081	0.2607249	5.34E-20	1.11E-15	0.731	0.738
MACO1	1.197898	0.2605047	1.28E-13	2.67E-09	0.483	0.452
PSMD2	1.197498	0.2600229	2.00E-23	4.16E-19	0.977	0.983

LAMA2	1.197394	0.2598979	4.18E-15	8.71E-11	0.4	0.328
PVR	1.197356	0.2598518	3.49E-13	7.27E-09	0.603	0.607
COL13A1	1.197291	0.2597739	5.08E-12	1.06E-07	0.459	0.423
CLCN7	1.197213	0.2596795	7.35E-11	1.53E-06	0.448	0.422
KCNE4	1.196953	0.259367	0.0004357	1	0.5	0.546
ZFC3H1	1.196666	0.2590209	1.64E-12	3.42E-08	0.44	0.39
POLR2A	1.196235	0.2585004	5.76E-14	1.20E-09	0.795	0.832
B4GALT4	1.196047	0.2582735	1.48E-11	3.09E-07	0.491	0.469
SRRM2	1.196035	0.2582601	1.21E-21	2.52E-17	0.978	0.971
SCAMP1	1.195876	0.2580677	3.15E-16	6.57E-12	0.533	0.502
SLC35A3	1.195751	0.2579167	1.19E-15	2.47E-11	0.472	0.414
SELENOI	1.195635	0.2577772	7.35E-15	1.53E-10	0.416	0.352
HACD2	1.195416	0.2575126	5.07E-10	1.06E-05	0.547	0.553
ITPR2	1.195234	0.257293	7.49E-13	1.56E-08	0.419	0.362
SPEN	1.19504	0.2570585	9.00E-13	1.87E-08	0.525	0.502
IFI16	1.194841	0.256819	7.72E-12	1.61E-07	0.719	0.769
DHCR24	1.194583	0.2565073	7.88E-07	0.0164168	0.516	0.539
BCLAF1	1.194279	0.2561399	7.64E-18	1.59E-13	0.837	0.86
MIR503HG	1.194131	0.2559608	0.0049678	1	0.362	0.375
EIF4G3	1.193799	0.2555604	8.91E-19	1.86E-14	0.717	0.734
CHID1	1.193793	0.2555524	3.36E-12	7.01E-08	0.517	0.505
NECTIN3	1.193055	0.254661	1.35E-10	2.81E-06	0.438	0.402
JAM3	1.192625	0.2541403	1.89E-13	3.93E-09	0.488	0.45
PSENEN	1.192167	0.2535858	2.99E-14	6.23E-10	0.509	0.476
SMARCA1	1.191742	0.2530725	9.80E-13	2.04E-08	0.597	0.618
SLC5A3	1.191447	0.2527152	4.82E-08	0.0010051	0.519	0.536
PSMD1	1.19133	0.2525735	8.43E-18	1.76E-13	0.862	0.908
SEC11C	1.191234	0.252457	8.54E-09	0.000178	0.583	0.623
WASHC4	1.190991	0.2521622	3.99E-13	8.31E-09	0.59	0.604
ALG8	1.190986	0.2521565	4.95E-16	1.03E-11	0.442	0.374
RSRP1	1.190868	0.252014	3.40E-08	0.0007084	0.531	0.544
ITPRIP	1.190294	0.2513177	3.80E-12	7.91E-08	0.544	0.534
TMEM45A	1.190208	0.2512143	6.54E-11	1.36E-06	0.465	0.444
LTN1	1.190066	0.2510414	7.35E-14	1.53E-09	0.497	0.465
FZD2	1.190011	0.250975	2.88E-09	6.00E-05	0.461	0.451
SNX14	1.189615	0.2504945	7.33E-15	1.53E-10	0.424	0.362
FNDC3B	1.189465	0.2503124	1.89E-16	3.94E-12	0.852	0.903
ANXA6	1.189405	0.2502406	3.67E-19	7.64E-15	0.94	0.961
GPX7	1.189291	0.250102	1.20E-11	2.51E-07	0.686	0.73

Cluster 5 of Integrated massives

Gene	Average fold-change	Average fold-change, log2	p-value	p_val_adj<br>Adjusted p-value, based on bonferroni correction	pct.1<br>The percentage of cells where the feature is detected in the first group	pct.2<br>The percentage of cells where the feature is detected in the second group
CD36	12.12704	3.6001555	3.23E-70	6.73E-66	0.965	0.538

PPP1R14A	8.852649	3.1461092	6.54E-100	1.36E-95	0.956	0.277
SYNPO2	5.763995	2.5270691	3.70E-86	7.71E-82	0.991	0.382
ADGRF5	5.438296	2.4431547	4.30E-229	8.96E-225	0.965	0.097
NDUFA4L2	5.086081	2.3465544	2.32E-80	4.84E-76	0.92	0.303
TINAGL1	5.012999	2.325674	7.33E-71	1.53E-66	0.982	0.542
ACTA2	4.891143	2.2901717	2.97E-57	6.20E-53	0.991	0.941
MYLK	4.462647	2.1578996	3.76E-64	7.83E-60	1	0.842
ACTG2	4.349452	2.1208335	1.25E-228	2.61E-224	0.664	0.039
HLA-B	4.324763	2.1126211	4.78E-67	9.95E-63	1	0.87
COL4A1	4.281161	2.098002	1.86E-68	3.88E-64	1	0.988
IGFBP5	4.074153	2.0265001	4.29E-38	8.94E-34	0.973	0.849
NES	3.961706	1.9861217	6.87E-126	1.43E-121	0.956	0.193
FABP4	3.763663	1.9121373	3.71E-103	7.72E-99	0.664	0.093
COL4A2	3.737858	1.9022118	8.83E-72	1.84E-67	1	0.994
C11orf96	3.713531	1.8927915	1.98E-60	4.13E-56	0.912	0.416
POSTN	3.605465	1.8501853	1.38E-38	2.88E-34	0.991	0.968
ACAN	3.595008	1.8459951	4.95E-118	1.03E-113	0.761	0.111
MCAM	3.513819	1.8130398	5.03E-87	1.05E-82	0.956	0.289
ITGA1	3.466899	1.793646	1.27E-73	2.64E-69	0.982	0.466
PPME1	3.437976	1.7815596	2.35E-49	4.89E-45	1	0.887
EDIL3	2.978888	1.5747737	1.10E-111	2.30E-107	0.956	0.212
IGFBP7	2.913738	1.5428712	2.74E-54	5.70E-50	1	0.999
RGS16	2.7028	1.434455	2.41E-64	5.02E-60	0.655	0.145
CTSD	2.63516	1.3978907	1.24E-53	2.58E-49	1	0.917
COL18A1	2.602124	1.3796896	1.44E-69	2.99E-65	0.903	0.318
AEBP1	2.55673	1.3542999	1.35E-51	2.81E-47	0.982	0.811
FHL1	2.52201	1.3345743	2.48E-34	5.18E-30	0.929	0.723
HLA-A	2.436442	1.2847761	2.28E-49	4.76E-45	1	0.907
MYH11	2.369019	1.2442898	1.86E-287	3.87E-283	0.522	0.016
CCN2	2.362473	1.2402979	1.19E-35	2.47E-31	0.991	0.996
CRYAB	2.360064	1.2388258	1.02E-22	2.13E-18	1	0.995
FILIP1L	2.35435	1.2353288	1.61E-41	3.35E-37	0.929	0.678
CALD1	2.343674	1.2287722	1.57E-47	3.26E-43	1	1
B2M	2.244211	1.1662085	1.32E-47	2.75E-43	1	1
KCTD20	2.208531	1.1430873	1.35E-34	2.82E-30	0.982	0.887
CSRP2	2.184948	1.1275991	6.40E-29	1.33E-24	0.761	0.41
JAG1	2.183857	1.1268781	8.79E-59	1.83E-54	0.85	0.295
TP53I11	2.1407	1.0980829	5.92E-39	1.23E-34	0.92	0.605
A2M	2.132333	1.0924325	2.39E-69	4.98E-65	0.796	0.21
GPX3	2.126679	1.0886026	3.49E-22	7.27E-18	0.779	0.516
PDGFA	2.112279	1.0788006	4.71E-68	9.81E-64	0.805	0.233
RARRES2	2.107867	1.0757839	3.73E-42	7.77E-38	0.832	0.358
COL5A3	2.067665	1.0480023	8.63E-48	1.80E-43	0.752	0.254
SRGN	2.065221	1.0462963	1.77E-28	3.69E-24	0.832	0.531
TAGLN	2.045708	1.0326001	3.59E-36	7.49E-32	1	1
ENAH	2.041582	1.0296873	2.12E-34	4.43E-30	1	0.95
MGP	2.03795	1.0271187	2.88E-09	6.00E-05	0.664	0.502
THBS1	2.025472	1.018258	5.59E-35	1.16E-30	1	0.996
MAF	2.004858	1.0034998	3.86E-36	8.04E-32	0.752	0.335
HLA-C	2.001509	1.0010878	1.34E-31	2.78E-27	0.991	0.954

MYL9	1.991213	0.9936475	3.92E-49	8.17E-45	1	0.999
EPAS1	1.978274	0.9842423	3.05E-38	6.36E-34	0.903	0.592
ARID5B	1.955694	0.9676808	1.45E-36	3.01E-32	0.982	0.947
EFHD1	1.93635	0.9533395	4.33E-63	9.03E-59	0.788	0.225
CPE	1.934871	0.9522374	2.19E-63	4.56E-59	0.805	0.235
ANGPTL4	1.930605	0.9490528	5.42E-27	1.13E-22	0.796	0.444
DSTN	1.920667	0.9416077	4.30E-45	8.96E-41	1	1
PLAC9	1.913606	0.9362941	7.83E-33	1.63E-28	0.956	0.824
BGN	1.896207	0.9231164	4.62E-39	9.63E-35	1	0.985
CXCL12	1.875683	0.9074161	3.57E-22	7.44E-18	0.593	0.261
NID1	1.875122	0.9069844	2.34E-30	4.87E-26	0.912	0.656
CAV1	1.857891	0.893666	4.50E-33	9.38E-29	1	0.994
COBLL1	1.852841	0.8897387	9.05E-38	1.89E-33	0.894	0.521
ARHGDIB	1.847483	0.8855608	3.11E-51	6.48E-47	0.779	0.249
LPP	1.84468	0.8833708	2.40E-35	5.01E-31	1	0.876
MYO1B	1.835043	0.8758136	1.59E-32	3.32E-28	0.982	0.835
ERRFI1	1.829202	0.8712144	1.64E-32	3.41E-28	0.805	0.436
ID4	1.822525	0.865939	5.45E-32	1.14E-27	0.823	0.42
FOS	1.794966	0.8439565	3.52E-15	7.32E-11	0.92	0.821
C1orf198	1.786454	0.8370988	5.71E-37	1.19E-32	0.982	0.862
CRISPLD2	1.778467	0.8306346	6.05E-36	1.26E-31	0.814	0.378
IFITM3	1.776481	0.8290223	4.26E-33	8.88E-29	1	0.995
GLDN	1.773294	0.826432	3.25E-133	6.78E-129	0.354	0.018
ZEB2	1.766905	0.8212248	4.17E-31	8.69E-27	0.832	0.493
CNN1	1.761065	0.8164484	7.35E-19	1.53E-14	0.876	0.68
WFDC1	1.741962	0.8007135	9.21E-253	1.92E-248	0.602	0.026
MAP1B	1.739385	0.798577	2.27E-31	4.73E-27	1	0.998
VEGFA	1.727648	0.7888089	1.83E-10	3.82E-06	0.823	0.717
PDLIM1	1.723829	0.7856168	1.45E-28	3.03E-24	0.938	0.771
CAV2	1.720926	0.7831853	1.04E-28	2.16E-24	0.965	0.867
ESAM	1.706241	0.7708216	0	0	0.531	0.014
LBH	1.705077	0.7698371	2.88E-17	6.01E-13	0.956	0.782
FAM107B	1.703059	0.7681288	3.24E-49	6.74E-45	0.637	0.167
APOE	1.690418	0.7573796	4.54E-29	9.46E-25	0.469	0.135
IGFBP2	1.689313	0.756437	1.45E-205	3.01E-201	0.469	0.019
MRVI1	1.688981	0.756153	7.03E-35	1.46E-30	0.779	0.352
INPP4B	1.687783	0.7551293	4.00E-86	8.34E-82	0.558	0.076
EDN1	1.684236	0.7520941	1.01E-19	2.11E-15	0.478	0.192
LMOD1	1.6787	0.747344	2.41E-55	5.03E-51	0.69	0.174
SDC2	1.654159	0.7260975	5.38E-19	1.12E-14	0.876	0.818
CRIM1	1.647678	0.7204343	5.81E-27	1.21E-22	1	0.993
NR2F2	1.647473	0.7202549	3.88E-25	8.09E-21	0.991	0.974
GNG11	1.637852	0.7118047	2.70E-12	5.63E-08	0.929	0.827
NID2	1.627992	0.7030939	1.48E-18	3.07E-14	0.85	0.696
NOTCH3	1.62463	0.7001112	6.98E-24	1.46E-19	0.876	0.578
C4orf3	1.623519	0.6991241	8.89E-20	1.85E-15	0.991	0.961
LOXL2	1.610535	0.6875402	1.13E-24	2.36E-20	1	0.996
VCL	1.607271	0.6846128	4.49E-28	9.35E-24	0.982	0.962
BNIP3	1.605945	0.6834226	8.84E-12	1.84E-07	0.912	0.887
SERTAD4-AS	1.602011	0.679884	1.40E-30	2.92E-26	0.558	0.193

PLN	1.601558	0.6794761	8.34E-267	1.74E-262	0.487	0.015
FLNA	1.59897	0.6771432	4.53E-31	9.45E-27	1	1
GMFG	1.597231	0.6755733	6.49E-197	1.35E-192	0.513	0.025
PDGFRB	1.595379	0.6738992	1.30E-20	2.70E-16	0.938	0.729
CREB5	1.588186	0.66738	2.67E-153	5.56E-149	0.504	0.032
IL7R	1.586727	0.6660543	5.72E-15	1.19E-10	0.708	0.46
STK38L	1.573651	0.654116	8.66E-19	1.80E-14	0.796	0.555
ACTN4	1.571792	0.6524105	1.50E-29	3.13E-25	1	0.996
PRRX1	1.559972	0.6415199	1.53E-21	3.18E-17	0.991	0.984
LAMC1	1.547806	0.6302246	3.60E-20	7.50E-16	0.982	0.954
PDE3A	1.546273	0.6287948	5.08E-29	1.06E-24	0.628	0.248
AOC3	1.543192	0.6259176	2.93E-294	6.10E-290	0.496	0.014
ADM	1.540216	0.6231331	1.31E-09	2.72E-05	0.805	0.722
ADAMTS5	1.539745	0.6226911	2.17E-23	4.53E-19	0.69	0.335
STOM	1.532569	0.6159518	4.73E-16	9.85E-12	0.823	0.652
PALLD	1.526294	0.6100327	1.64E-17	3.43E-13	0.965	0.879
FAM43A	1.525735	0.6095043	3.75E-07	0.0078168	0.673	0.533
PSAP	1.515258	0.5995635	1.44E-21	3.00E-17	0.991	0.959
NEXN	1.514767	0.5990961	6.67E-16	1.39E-11	0.982	0.906
GJA5	1.514183	0.5985399	1.16E-239	2.42E-235	0.531	0.021
ILK	1.513676	0.5980564	9.44E-24	1.97E-19	0.982	0.9
MYH9	1.513181	0.597585	8.18E-24	1.70E-19	1	0.999
CHCHD10	1.509704	0.5942661	1.98E-14	4.13E-10	0.885	0.791
KIAA0040	1.509042	0.5936332	3.68E-45	7.66E-41	0.593	0.153
ITGA3	1.508753	0.593357	4.25E-20	8.85E-16	0.779	0.5
RABGAP1	1.505404	0.5901506	6.09E-23	1.27E-18	0.726	0.39
ITGA7	1.503369	0.588199	1.92E-162	3.99E-158	0.531	0.034
LEPROT	1.503006	0.5878503	8.55E-16	1.78E-11	0.965	0.867
PLXND1	1.498927	0.5839305	5.21E-16	1.09E-11	0.956	0.847
SYTL2	1.4975	0.5825561	8.60E-98	1.79E-93	0.575	0.069
PODN	1.497371	0.5824316	2.61E-13	5.44E-09	0.69	0.501
SUSD2	1.495085	0.5802279	1.55E-31	3.23E-27	0.584	0.188
ENDOD1	1.489212	0.5745491	2.54E-20	5.29E-16	0.77	0.472
MSRB3	1.487654	0.573039	1.09E-20	2.27E-16	0.982	0.873
HLA-E	1.487353	0.5727468	1.16E-14	2.42E-10	0.956	0.889
TFPI	1.483904	0.5693982	1.29E-18	2.68E-14	0.637	0.333
LASP1	1.481792	0.5673427	1.82E-13	3.80E-09	0.973	0.961
COL15A1	1.481267	0.5668319	1.03E-07	0.0021422	0.513	0.332
ADAMTS2	1.479768	0.565371	3.52E-17	7.34E-13	0.982	0.946
H2AFJ	1.479477	0.5650875	3.66E-19	7.63E-15	0.965	0.905
KHDRBS3	1.474803	0.5605224	4.42E-17	9.22E-13	0.664	0.391
FARP1	1.474516	0.5602418	9.61E-19	2.00E-14	0.85	0.681
DAAM2	1.474507	0.5602323	6.62E-35	1.38E-30	0.575	0.177
ROCK2	1.471443	0.5572321	1.19E-20	2.48E-16	0.956	0.791
LZTS1	1.46823	0.5540782	2.76E-33	5.75E-29	0.619	0.209
CYGB	1.466191	0.5520728	5.26E-06	0.1096063	0.814	0.749
CARMN	1.464927	0.5508284	1.74E-09	3.62E-05	0.752	0.576
ITGB1	1.463174	0.5491017	3.17E-27	6.60E-23	1	1
NTN4	1.460463	0.5464263	7.52E-66	1.57E-61	0.496	0.074
KLF2	1.460007	0.5459754	2.39E-09	4.97E-05	0.903	0.838

AC109479.1	1.457978	0.5439689	3.67E-152	7.65E-148	0.469	0.028
CDH6	1.454206	0.5402313	2.50E-32	5.20E-28	0.513	0.146
SORT1	1.453686	0.5397159	2.99E-23	6.23E-19	0.664	0.311
TRPV2	1.452447	0.5384858	7.85E-25	1.63E-20	0.602	0.235
BMERB1	1.451645	0.5376891	5.70E-21	1.19E-16	0.69	0.373
NEDD9	1.447284	0.5333484	1.74E-16	3.62E-12	0.664	0.395
SLC20A2	1.447062	0.5331271	2.77E-17	5.76E-13	0.761	0.518
VGLL3	1.443203	0.5292746	3.41E-14	7.11E-10	0.841	0.688
TBC1D1	1.443084	0.529155	1.45E-21	3.03E-17	0.832	0.509
CNN3	1.439713	0.5257813	6.38E-22	1.33E-17	1	0.955
ADGRE5	1.438832	0.524898	1.24E-10	2.59E-06	0.805	0.747
PAPPA	1.436537	0.5225951	0.006134	1	0.69	0.681
LTBP1	1.434529	0.5205769	1.06E-10	2.20E-06	0.558	0.331
CYB5R3	1.434318	0.5203649	1.25E-20	2.61E-16	1	0.997
ARSJ	1.427145	0.5131316	3.07E-25	6.39E-21	0.549	0.208
ANKRD12	1.426437	0.5124161	2.15E-13	4.47E-09	0.885	0.75
BNIP3L	1.426117	0.5120919	1.39E-15	2.90E-11	0.956	0.853
PELO	1.423863	0.5098099	9.50E-14	1.98E-09	0.823	0.691
MEF2C	1.421914	0.5078342	1.30E-14	2.71E-10	0.735	0.504
GYPC	1.420509	0.5064079	2.19E-11	4.57E-07	0.85	0.741
FERMT2	1.417723	0.503576	5.01E-11	1.04E-06	0.982	0.954
SH3BGRL	1.417223	0.5030668	9.88E-16	2.06E-11	0.885	0.668
RHOBTB3	1.417155	0.5029976	2.32E-11	4.84E-07	0.929	0.891
TPM4	1.413223	0.4989894	2.98E-21	6.22E-17	1	1
ADAMTS12	1.412558	0.4983106	1.30E-12	2.71E-08	0.628	0.399
AOPEP	1.412451	0.4982005	1.99E-13	4.16E-09	0.814	0.652
DLC1	1.403899	0.4894393	3.25E-14	6.78E-10	0.982	0.913
MYO1E	1.40298	0.4884945	4.10E-15	8.55E-11	0.832	0.706
ADAM9	1.398959	0.4843536	2.63E-12	5.47E-08	0.903	0.835
FBXO32	1.397576	0.4829268	2.02E-10	4.21E-06	0.735	0.534
CYP26B1	1.396448	0.4817615	1.98E-109	4.13E-105	0.31	0.016
COX20	1.391027	0.4761505	1.68E-11	3.51E-07	0.903	0.829
CCDC50	1.389005	0.4740519	6.41E-12	1.34E-07	0.903	0.817
TBL1XR1	1.388154	0.4731674	2.48E-14	5.17E-10	0.85	0.671
CFL2	1.383987	0.4688307	5.74E-11	1.20E-06	0.929	0.898
PKD2	1.381566	0.4663049	3.80E-12	7.92E-08	0.894	0.788
NPC2	1.380329	0.465012	4.23E-10	8.82E-06	0.991	0.983
JUND	1.378881	0.4634977	2.56E-10	5.33E-06	0.894	0.875
LAMA4	1.37799	0.4625652	6.84E-08	0.0014257	0.876	0.762
SGIP1	1.375799	0.4602698	3.08E-177	6.41E-173	0.46	0.022
C1R	1.375733	0.4602009	2.82E-12	5.87E-08	0.584	0.347
MXRA7	1.374969	0.459399	6.00E-12	1.25E-07	0.991	0.978
LMCD1	1.374687	0.459103	8.04E-08	0.0016755	0.788	0.649
EPS8	1.374521	0.4589291	2.56E-12	5.33E-08	0.956	0.934
PRXL2A	1.374389	0.45879	1.79E-29	3.72E-25	0.487	0.142
BST2	1.374282	0.4586786	1.71E-56	3.56E-52	0.434	0.064
STAT1	1.373741	0.4581101	8.45E-10	1.76E-05	0.841	0.692
SNTB1	1.372521	0.4568285	5.22E-22	1.09E-17	0.478	0.171
VMP1	1.372475	0.4567804	9.29E-13	1.94E-08	0.965	0.891
FAM241A	1.370384	0.4545798	2.80E-20	5.84E-16	0.575	0.254

AMIGO2	1.369637	0.4537936	6.79E-07	0.0141388	0.85	0.794
METRNL	1.367337	0.4513685	1.52E-07	0.0031623	0.929	0.932
IFITM2	1.366388	0.4503669	1.57E-14	3.27E-10	1	0.989
SPOCD1	1.365924	0.4498774	3.36E-11	7.00E-07	0.858	0.755
CHN1	1.363949	0.4477898	1.74E-16	3.62E-12	0.549	0.257
PALM2-AKA	1.363164	0.4469593	1.36E-12	2.83E-08	0.956	0.902
MACF1	1.361854	0.4455721	3.02E-11	6.29E-07	0.965	0.93
H1FX	1.361125	0.4447996	9.35E-09	0.0001947	0.726	0.596
PRKAG2	1.359727	0.4433166	1.18E-20	2.47E-16	0.628	0.3
PDLIM3	1.359279	0.4428413	9.35E-07	0.0194828	0.46	0.3
GJC1	1.358608	0.4421292	3.36E-24	7.00E-20	0.549	0.2
CCN1	1.358103	0.441593	2.94E-17	6.13E-13	0.991	0.999
CCDC107	1.355957	0.4393116	8.64E-10	1.80E-05	0.832	0.765
MYL6	1.353886	0.4371058	1.05E-17	2.20E-13	1	1
LHFPL6	1.352641	0.4357788	6.44E-16	1.34E-11	0.912	0.686
ARHGAP24	1.350265	0.4332423	1.12E-09	2.33E-05	0.752	0.59
CLTB	1.349475	0.4323981	1.14E-12	2.37E-08	0.947	0.922
MFGE8	1.34933	0.4322437	7.64E-11	1.59E-06	0.991	0.969
NQO1	1.348038	0.4308614	0.0013393	1	0.903	0.922
SEPTIN7	1.34773	0.4305312	2.94E-12	6.13E-08	1	0.971
AC113383.1	1.346982	0.4297308	1.65E-83	3.43E-79	0.389	0.036
SNX18	1.345367	0.4280003	4.88E-12	1.02E-07	0.575	0.348
INAFM2	1.342279	0.4246842	3.62E-30	7.54E-26	0.469	0.132
COX7A1	1.341524	0.4238731	3.26E-11	6.80E-07	0.982	0.966
A1BG	1.341421	0.4237619	7.53E-20	1.57E-15	0.566	0.238
PEA15	1.339325	0.4215063	2.22E-15	4.62E-11	0.982	0.978
LACTB	1.338221	0.4203159	5.45E-09	0.0001135	0.858	0.716
NEAT1	1.337376	0.4194048	3.24E-05	0.6759182	1	1
ATP2A2	1.336463	0.4184199	7.26E-11	1.51E-06	0.965	0.923
MBNL1	1.33318	0.4148713	9.65E-10	2.01E-05	0.956	0.927
LCP1	1.333007	0.4146842	1.15E-29	2.40E-25	0.354	0.077
LY6K	1.331815	0.4133933	3.59E-12	7.47E-08	0.451	0.22
COMMD7	1.331012	0.4125236	2.62E-09	5.46E-05	0.779	0.678
KCNMB1	1.329902	0.4113195	7.67E-253	1.60E-248	0.381	0.009
MYO1D	1.329295	0.4106609	1.12E-18	2.32E-14	0.637	0.314
CHPF	1.328582	0.4098875	3.12E-11	6.51E-07	0.938	0.883
CHSY3	1.328496	0.4097942	4.54E-19	9.46E-15	0.478	0.184
DEGS1	1.324467	0.4054114	7.70E-13	1.60E-08	0.982	0.87
UGP2	1.324099	0.4050112	3.35E-10	6.97E-06	0.929	0.884
ETS1	1.322447	0.4032095	9.96E-10	2.07E-05	0.796	0.696
GPR183	1.320534	0.401121	8.59E-101	1.79E-96	0.31	0.018
LRRFIP1	1.319942	0.4004744	4.04E-08	0.0008419	0.973	0.94
BTG1	1.319335	0.3998113	3.16E-09	6.58E-05	0.796	0.686
EBF1	1.318157	0.3985217	3.42E-15	7.14E-11	0.611	0.328
DKK3	1.317564	0.3978732	4.27E-07	0.008893	0.761	0.626
HES4	1.317507	0.3978105	2.17E-10	4.52E-06	0.619	0.4
LRRC8A	1.316281	0.3964673	1.12E-10	2.33E-06	0.796	0.66
ACTN1	1.315521	0.3956338	2.82E-16	5.89E-12	1	0.999
CYFIP2	1.31245	0.3922625	3.34E-56	6.96E-52	0.372	0.048
AC092807.3	1.311774	0.3915191	5.14E-09	0.000107	0.85	0.717

SLC2A1	1.310429	0.3900391	8.57E-10	1.79E-05	0.673	0.459
SPECC1	1.308796	0.3882399	4.80E-18	1.00E-13	0.566	0.249
JUN	1.307579	0.3868983	0.0005204	1	0.867	0.842
TBX2	1.305888	0.3850316	1.81E-07	0.003761	0.602	0.425
PLEKHA2	1.30562	0.3847349	2.48E-14	5.17E-10	0.593	0.322
ASAH1	1.304543	0.3835444	1.41E-09	2.93E-05	0.823	0.706
MAP3K7CL	1.303751	0.3826679	1.68E-15	3.50E-11	0.451	0.19
TCF7L1	1.301991	0.3807198	1.58E-14	3.29E-10	0.673	0.394
FAT1	1.301563	0.3802455	1.48E-08	0.0003089	0.823	0.784
GPI	1.301543	0.3802233	1.45E-11	3.02E-07	0.973	0.894
TES	1.300762	0.3793571	3.34E-10	6.97E-06	0.841	0.74
RBPMS	1.29874	0.3771127	3.84E-10	8.00E-06	0.832	0.717
PLPPR4	1.298332	0.3766589	4.59E-47	9.56E-43	0.319	0.042
WSB1	1.297548	0.3757877	3.83E-08	0.0007975	0.858	0.716
GLIPR1	1.297544	0.3757834	1.07E-09	2.24E-05	0.956	0.903
FADS3	1.297361	0.3755796	2.22E-10	4.62E-06	0.761	0.606
INKA2	1.296661	0.3748009	1.69E-14	3.53E-10	0.46	0.204
MPRIP	1.296523	0.3746476	1.85E-12	3.86E-08	0.991	0.95
TACC1	1.296182	0.3742681	9.30E-10	1.94E-05	0.823	0.682
TNS3	1.294409	0.372294	8.44E-11	1.76E-06	0.407	0.198
PAWR	1.294323	0.3721972	3.10E-09	6.46E-05	0.876	0.777
IFFO2	1.293988	0.3718245	2.22E-17	4.62E-13	0.434	0.164
CPQ	1.292693	0.3703793	1.33E-09	2.77E-05	0.69	0.519
SORBS2	1.29252	0.3701868	4.84E-21	1.01E-16	0.327	0.087
FAM91A1	1.291365	0.3688972	2.43E-07	0.0050668	0.699	0.562
FNIP2	1.291244	0.3687616	8.81E-08	0.0018362	0.664	0.517
CSPG4	1.290531	0.3679645	6.37E-10	1.33E-05	0.54	0.334
GBE1	1.289922	0.3672836	1.63E-10	3.40E-06	0.752	0.562
TPM2	1.287284	0.36433	6.79E-06	0.1414525	1	1
SOBP	1.286449	0.3633944	1.23E-13	2.57E-09	0.504	0.253
BST1	1.285727	0.3625839	1.10E-08	0.00023	0.708	0.55
PSME2	1.285664	0.3625133	5.61E-05	1	0.77	0.719
SERTAD4	1.284499	0.3612062	3.76E-40	7.84E-36	0.319	0.049
PRUNE2	1.28389	0.3605219	1.51E-16	3.16E-12	0.372	0.13
TAOK3	1.28351	0.360094	5.41E-09	0.0001127	0.788	0.683
ATF3	1.281854	0.3582322	1.15E-08	0.0002386	0.46	0.267
SSB	1.281766	0.3581327	0.0001274	1	0.903	0.869
PGK1	1.281469	0.3577983	1.15E-10	2.40E-06	0.991	0.992
MAP7D3	1.28081	0.357057	1.82E-10	3.78E-06	0.796	0.597
CYSTM1	1.278197	0.3541105	3.19E-08	0.0006646	0.885	0.814
TRPC6	1.277251	0.3530422	1.02E-258	2.13E-254	0.319	0.005
FRMD4A	1.277038	0.3528013	2.94E-08	0.0006122	0.805	0.679
KIAA1324L	1.277001	0.3527599	9.19E-17	1.92E-12	0.336	0.108
RND3	1.276075	0.3517128	3.51E-07	0.0073102	0.912	0.891
CCL3	1.275508	0.3510719	1.83E-10	3.81E-06	0.257	0.091
PRKG1	1.275486	0.3510473	3.86E-09	8.05E-05	0.611	0.425
IL1R1	1.275337	0.350878	1.44E-11	3.00E-07	0.637	0.416
TGFBR1	1.274479	0.349908	4.82E-09	0.0001005	0.566	0.363
MALAT1	1.273477	0.3487724	1.52E-08	0.0003176	1	1
ITIH5	1.272982	0.3482119	3.07E-36	6.39E-32	0.345	0.061

PCGF5	1.271406	0.3464243	8.34E-09	0.0001737	0.619	0.417
FOXO3	1.271154	0.3461387	1.03E-07	0.0021393	0.77	0.622
CCDC3	1.27115	0.3461346	4.10E-56	8.55E-52	0.327	0.037
TNS1	1.270133	0.3449799	2.94E-07	0.0061299	0.92	0.939
H1F0	1.268376	0.3429826	2.19E-11	4.55E-07	0.504	0.269
MYL12A	1.268037	0.3425964	6.27E-07	0.0130733	1	0.999
DUSP1	1.267562	0.3420562	1.17E-07	0.0024424	0.973	0.954
SEPTIN11	1.266856	0.3412525	9.38E-08	0.0019538	1	0.988
RERE	1.266612	0.3409749	1.22E-08	0.0002533	0.681	0.536
LPIN2	1.266412	0.3407469	2.62E-13	5.47E-09	0.735	0.475
HAPLN3	1.263656	0.3376039	3.90E-08	0.0008133	0.487	0.307
TRAM2	1.262509	0.3362933	2.67E-10	5.56E-06	0.956	0.925
PMP22	1.261951	0.3356562	0.0003379	1	0.805	0.758
CPNE2	1.261762	0.3354394	1.98E-16	4.13E-12	0.54	0.252
ABHD4	1.261356	0.3349756	4.12E-11	8.59E-07	0.566	0.325
VASP	1.261235	0.3348366	4.11E-08	0.000856	0.92	0.861
CALM2	1.260739	0.3342701	9.15E-10	1.91E-05	1	1
UBE2E2	1.260586	0.334095	1.89E-07	0.0039407	0.637	0.47
HTATIP2	1.260367	0.3338434	1.08E-10	2.25E-06	0.602	0.371
PEAK1	1.260124	0.3335658	9.21E-09	0.000192	0.823	0.712
CD248	1.260021	0.3334472	2.91E-07	0.0060559	0.973	0.986
C3orf70	1.259925	0.3333377	6.37E-47	1.33E-42	0.363	0.054
MAP2K1	1.258232	0.3313984	1.16E-07	0.0024252	0.743	0.609
EPB41L2	1.257901	0.3310187	9.47E-06	0.1973664	0.708	0.604
CCL2	1.257804	0.3309073	0.0007452	1	0.434	0.326
FGD4	1.257361	0.3303989	1.54E-30	3.21E-26	0.319	0.061
NUAK1	1.257325	0.3303572	0.0004845	1	0.761	0.734
UTRN	1.257301	0.3303304	1.57E-05	0.3276896	0.867	0.792
TMEM45A	1.257279	0.3303043	1.37E-05	0.2858026	0.593	0.443
SLC9A3R2	1.256633	0.3295635	1.91E-06	0.0397277	0.566	0.438
ERO1A	1.255692	0.3284826	8.24E-07	0.0171583	0.788	0.705
ITGA5	1.255371	0.3281137	0.0001273	1	0.991	0.987
HDAC9	1.2552	0.3279171	6.33E-17	1.32E-12	0.389	0.136
LAMP2	1.254968	0.3276511	2.53E-07	0.0052741	0.92	0.847
ITGAV	1.254579	0.3272038	1.22E-06	0.0255	0.903	0.877
SORBS1	1.252267	0.3245423	2.48E-65	5.17E-61	0.345	0.035
MAP3K8	1.252015	0.3242514	8.45E-09	0.0001761	0.442	0.256
KITLG	1.251932	0.3241563	2.20E-08	0.0004593	0.575	0.38
GSN	1.250372	0.3223569	1.01E-05	0.2108503	0.858	0.79
PPARG	1.249961	0.3218831	3.70E-40	7.70E-36	0.425	0.083
ACTR3	1.24964	0.321512	3.62E-11	7.53E-07	1	0.984
ST5	1.248878	0.320633	3.43E-10	7.15E-06	0.549	0.328
SMIM3	1.247975	0.3195889	6.15E-08	0.0012805	0.487	0.309
MAP2K3	1.246986	0.3184451	3.92E-05	0.817555	0.92	0.901
MYOCD	1.246947	0.3184	2.96E-09	6.17E-05	0.354	0.166
LGI4	1.246796	0.318225	1.22E-140	2.55E-136	0.301	0.011
ADAP2	1.246404	0.317772	1.81E-131	3.78E-127	0.31	0.013
ATP6V1B2	1.246357	0.3177177	1.38E-06	0.0286555	0.575	0.44
HSPA2	1.245908	0.3171972	4.46E-15	9.29E-11	0.381	0.14
MBNL1-AS1	1.24428	0.3153112	5.27E-13	1.10E-08	0.442	0.204

PNRC1	1.243732	0.3146761	9.26E-07	0.0193007	0.681	0.561
AGAP1	1.243543	0.3144561	2.76E-11	5.76E-07	0.487	0.25
FAM162A	1.243453	0.3143517	2.06E-08	0.0004282	0.743	0.554
DYNLT3	1.242017	0.312685	0.0007208	1	0.611	0.553
CTNNA1	1.241894	0.3125421	8.49E-07	0.0176889	0.947	0.907
TPP1	1.240951	0.3114458	3.98E-06	0.0829112	0.708	0.584
LINC01197	1.240516	0.3109405	4.72E-153	9.82E-149	0.336	0.013
ARHGAP29	1.238867	0.3090215	4.30E-07	0.0089574	0.469	0.302
ATP8B1	1.238566	0.3086711	4.39E-08	0.0009146	0.761	0.572
DAB2IP	1.237456	0.3073773	1.28E-11	2.66E-07	0.372	0.16
C1S	1.237232	0.3071161	8.75E-07	0.0182376	0.584	0.439
PDLIM5	1.235736	0.3053703	5.17E-07	0.0107751	0.965	0.934
NOG	1.233655	0.3029388	2.89E-31	6.02E-27	0.319	0.059
COL6A2	1.231813	0.3007833	5.38E-10	1.12E-05	1	1
UBE2E1	1.231594	0.3005266	3.42E-05	0.7119932	0.619	0.498
MECP2	1.230932	0.2997511	1.95E-08	0.0004069	0.699	0.511
SPTSSA	1.230749	0.2995367	1.38E-06	0.0287233	0.717	0.565
PTPRJ	1.230651	0.2994213	6.45E-11	1.34E-06	0.487	0.258
SLC2A3	1.230603	0.299365	4.27E-05	0.8903279	0.681	0.581
FGFR1OP2	1.230338	0.2990551	9.81E-07	0.0204407	0.796	0.68
PDE1C	1.23031	0.2990219	1.06E-06	0.0221031	0.593	0.432
APOLD1	1.230072	0.2987425	5.14E-16	1.07E-11	0.283	0.083
ENO2	1.230032	0.2986957	3.96E-07	0.0082474	0.487	0.306
BACE2	1.229828	0.298456	1.02E-09	2.12E-05	0.637	0.421
MAML2	1.228966	0.2974454	9.01E-05	1	0.549	0.456
TMEM165	1.228109	0.2964384	1.95E-05	0.4059389	0.912	0.885
C2CD2	1.227685	0.2959408	1.81E-05	0.3772438	0.513	0.371
PLOD1	1.227545	0.2957762	5.95E-06	0.1239823	0.85	0.816
GADD45B	1.226698	0.2947804	0.0008072	1	0.912	0.875
RNASEH2C	1.22664	0.2947113	4.77E-06	0.0994083	0.876	0.802
RBM24	1.226567	0.2946255	1.36E-16	2.84E-12	0.327	0.102
OGFRL1	1.226374	0.2943991	4.05E-07	0.0084472	0.619	0.456
ARHGEF12	1.22583	0.2937588	2.11E-05	0.4389476	0.717	0.628
FNDC3B	1.225793	0.2937159	0.0002133	1	0.92	0.899
DAD1	1.225412	0.2932673	0.0001134	1	1	0.996
PLEKHO1	1.225137	0.2929427	1.00E-05	0.2088646	0.885	0.813
HIF1A	1.224936	0.2927062	1.11E-06	0.0230977	1	0.999
PHF11	1.224727	0.2924604	1.99E-08	0.0004156	0.531	0.336
IL4R	1.224265	0.2919156	2.76E-09	5.74E-05	0.628	0.404
STARD4	1.22416	0.2917917	2.59E-05	0.5393299	0.584	0.461
HES1	1.223987	0.2915885	0.000482	1	0.885	0.897
SMAP2	1.22389	0.2914736	6.71E-10	1.40E-05	0.699	0.496
MINDY2	1.223678	0.2912243	5.51E-07	0.0114773	0.69	0.556
KIF13A	1.223494	0.2910066	1.24E-06	0.025931	0.584	0.436
TPI1	1.222127	0.2893941	7.98E-07	0.0166226	1	0.999
SH3RF1	1.220417	0.2873744	5.75E-06	0.1197873	0.752	0.698
LIF	1.219262	0.2860079	0.0056584	1	0.336	0.256
PXDN	1.21834	0.2849172	8.24E-07	0.0171733	0.973	0.967
ARHGAP1	1.21805	0.2845733	6.66E-06	0.138723	0.894	0.888
KANK2	1.215496	0.2815455	8.96E-06	0.1867476	0.867	0.795

SLCO3A1	1.215031	0.2809931	2.56E-09	5.33E-05	0.425	0.221
TRIOBP	1.214963	0.2809127	1.04E-06	0.0217264	0.858	0.781
HSPB8	1.214229	0.28004	4.11E-06	0.0855771	0.611	0.491
TMEM123	1.21413	0.2799228	4.42E-05	0.9202331	0.841	0.77
AKR1A1	1.214008	0.2797783	5.63E-07	0.0117312	0.673	0.536
CCDC81	1.213814	0.2795474	2.14E-32	4.46E-28	0.354	0.07
PHC2	1.213348	0.2789935	9.05E-06	0.1886459	0.602	0.465
CTSA	1.21319	0.2788051	0.0025329	1	0.947	0.887
NR2F1	1.213173	0.2787847	0.0040336	1	0.637	0.589
CD4	1.21266	0.2781747	8.10E-140	1.69E-135	0.283	0.01
PLS3	1.212615	0.2781213	3.45E-05	0.7179466	0.973	0.967
SERPING1	1.212338	0.2777915	1.25E-05	0.2614201	0.575	0.436
FNBP1	1.211662	0.2769877	1.27E-08	0.000264	0.584	0.376
PSME1	1.21154	0.2768416	0.003375	1	0.575	0.516
ZNF292	1.211117	0.276338	0.0008239	1	0.602	0.541
TIMP2	1.2103	0.2753653	1.20E-05	0.2509367	1	0.999
SERINC2	1.208244	0.2729117	0.0002084	1	0.637	0.56
LITAF	1.207782	0.2723596	2.17E-06	0.0451762	0.69	0.539
UPP1	1.206921	0.2713317	1.17E-05	0.244785	0.584	0.44
SELENOP	1.206384	0.2706893	8.91E-06	0.1856013	0.469	0.313
BDNF	1.205888	0.2700955	0.0003178	1	0.469	0.36
ITM2C	1.205842	0.2700404	0.0004344	1	0.69	0.606
SYNM	1.205799	0.2699892	2.72E-25	5.66E-21	0.31	0.067
ZYX	1.20559	0.2697399	4.93E-09	0.0001027	0.991	0.996
COA3	1.205088	0.269139	9.31E-07	0.0193974	0.735	0.591
FOSL2	1.204233	0.2681151	1.89E-06	0.0394675	0.805	0.687
PLAU	1.20335	0.2670565	0.0003178	1	0.372	0.241
ANXA6	1.202249	0.2657356	1.31E-05	0.2729861	0.973	0.96
KLF9	1.202028	0.2654703	0.0005033	1	0.407	0.307
LIMS1	1.200887	0.2641006	0.0027111	1	0.973	0.967
LIN7A	1.200348	0.2634522	5.60E-94	1.17E-89	0.274	0.015
TMEM119	1.200034	0.2630747	1.60E-05	0.3334558	0.469	0.328
YAP1	1.19962	0.2625776	1.31E-06	0.0272426	0.823	0.693
TAPBP	1.198687	0.2614549	5.91E-05	1	0.841	0.76
SRF	1.198645	0.2614042	1.73E-06	0.0360494	0.619	0.45
DDX5	1.198489	0.261217	4.40E-07	0.009163	0.991	0.982
COL25A1	1.197971	0.2605926	1.64E-83	3.41E-79	0.265	0.016
GPNMB	1.196844	0.2592351	9.73E-05	1	0.885	0.875
NDUFV2	1.196718	0.2590836	7.39E-05	1	0.929	0.885
KMT2E	1.19652	0.2588444	1.54E-05	0.3206026	0.752	0.65
MT1F	1.19599	0.2582051	9.74E-07	0.0203022	0.442	0.281
CDH13	1.19584	0.2580241	0.000132	1	0.726	0.651
PPP1R3B	1.195525	0.2576445	1.33E-07	0.0027686	0.381	0.208
SLC25A1	1.19467	0.256612	4.29E-06	0.0892759	0.858	0.797
CSRP1	1.193775	0.2555304	4.81E-05	1	1	0.99
HTRA1	1.193534	0.2552398	7.16E-06	0.149076	0.982	0.971
RAB23	1.193215	0.2548536	4.97E-07	0.0103581	0.584	0.413
SLC25A5	1.192831	0.2543897	3.17E-05	0.6603419	0.965	0.932
HLA-F	1.192825	0.2543827	1.96E-14	4.09E-10	0.31	0.1
PRKAB2	1.192687	0.2542153	1.77E-07	0.0036782	0.478	0.291

MCU	1.192648	0.2541688	1.46E-05	0.3035414	0.487	0.356
PTPRA	1.192469	0.2539523	1.23E-06	0.025543	0.726	0.596
JADE1	1.19239	0.2538562	0.0032224	1	0.655	0.619
BIRC2	1.192364	0.2538253	0.0003745	1	0.832	0.771
UBE2V1	1.192109	0.2535165	8.20E-05	1	0.973	0.945
HIVEP1	1.191927	0.2532958	3.25E-05	0.6775543	0.496	0.372
NREP	1.191922	0.2532899	0.0012188	1	0.779	0.759
FAP	1.191892	0.2532538	0.0003237	1	0.752	0.687
CALM1	1.191713	0.2530373	7.34E-07	0.0152945	0.991	0.996
MAP3K20	1.191584	0.2528803	5.03E-06	0.1047648	0.814	0.688
CTSZ	1.190716	0.2518292	0.0004106	1	0.903	0.885
PMEPA1	1.19058	0.2516643	0.0014804	1	0.611	0.535
NPC1	1.190367	0.2514061	0.0009718	1	0.451	0.352
VSIR	1.190231	0.2512413	1.77E-11	3.68E-07	0.398	0.179
AK4	1.19012	0.2511068	0.0002969	1	0.699	0.631
TMEM59	1.189541	0.2504045	0.0083333	1	0.876	0.874
